# Polymeric Nanoparticles for Drug Delivery in Osteoarthritis

**DOI:** 10.3390/pharmaceutics14122639

**Published:** 2022-11-29

**Authors:** Adriano P. Pontes, Tim J. M. Welting, Jaap Rip, Laura B. Creemers

**Affiliations:** 1 20Med Therapeutics B.V., 2333 BD Leiden, The Netherlands; 2 Laboratory for Experimental Orthopedics, Department of Orthopedic Surgery, Maastricht University Medical Center, 6229 HX Maastricht, The Netherlands; 3 Department of Orthopedics, University Medical Center Utrecht, 3584 CX Utrecht, The Netherlands

**Keywords:** nanoparticles, polymers, osteoarthritis, cartilage, therapeutics

## Abstract

Osteoarthritis (OA) is a degenerative musculoskeletal disorder affecting the whole synovial joint and globally impacts more than one in five individuals aged 40 and over, representing a huge socioeconomic burden. Drug penetration into and retention within the joints are major challenges in the development of regenerative therapies for OA. During the recent years, polymeric nanoparticles (PNPs) have emerged as promising drug carrier candidates due to their biodegradable properties, nanoscale structure, functional versatility, and reproducible manufacturing, which makes them particularly attractive for cartilage penetration and joint retention. In this review, we discuss the current development state of natural and synthetic PNPs for drug delivery and OA treatment. Evidence from in vitro and pre-clinical in vivo studies is used to show how disease pathology and key cellular pathways of joint inflammation are modulated by these nanoparticle-based therapies. Furthermore, we compare the biodegradability and surface modification of these nanocarriers in relation to the drug release profile and tissue targeting. Finally, the main challenges for nanoparticle delivery to the cartilage are discussed, as a function of disease state and physicochemical properties of PNPs such as size and surface charge.

## 1. Introduction

### 1.1. Osteoarthritis

Osteoarthritis (OA) is a degenerative disorder that involves damage to the articular cartilage and osseous overgrowth around the joints. The disease leads to severe pain, loss of joint function and impaired quality of life [1]. This arthritic condition can result from genetic predisposition and/or cumulative wear and tear on joint surfaces, although it has become gradually clear that OA is not only a mechanical problem [2]. Osteoarthritis is strongly linked with risk factors such as obesity and injury [3,4]. The global prevalence of knee OA in 2020 was 22.9% in individuals aged 40 and over, corresponding to 654.1 million individuals worldwide in this age range, and with a prevalence in females 1.69 times higher than in males [5].

Cartilage destruction is regarded as the hallmark of osteoarthritis [6]. The articular cartilage is a load bearing tissue which covers the end of bones in synovial joints, and chondrocytes are the sole cells residing in this tissue. They are dispersed in an extracellular matrix (ECM) consisting of collagen type II fibers and proteoglycans, together forming a highly dense and negatively charged meshwork [7]. Because it is an avascular and alymphatic tissue, the articular cartilage largely lacks self-healing capacity due to the poor blood supply and low metabolic rate [8]. Recent research supports that OA is a “whole joint” disease that also affects other structures in the synovial joint, leading to synovitis, degeneration of ligaments and menisci, inflammation of the infrapatellar fat pad, hypertrophy of the joint capsule, subchondral bone remodeling, and osteophyte formation [9,10].

**Figure 1:**
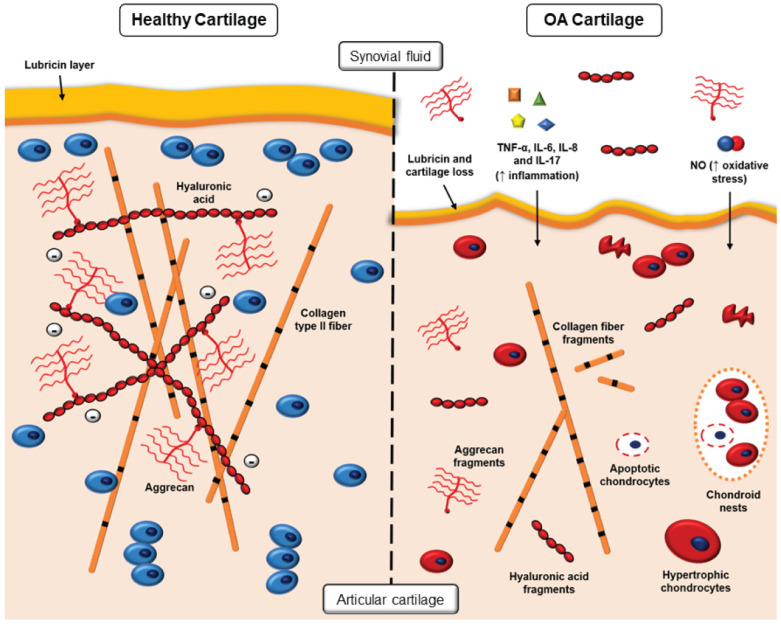
Healthy and osteoarthritic cartilage structure. In healthy cartilage, the fibrillar collagen network enriched in proteoglycans is a highly dense and negatively charged matrix. Osteoarthritis is a joint disorder involving the degeneration of articular cartilage, characterized by degradation of aggrecan proteoglycans by ADAMTS-4, loss and replacement of collagen type II by collagen type I, and increased apoptosis of chondrocytes; all these changes ultimately lead to the disturbance of cartilage homeostasis and loss of joint function. During OA, chondrocytes are exposed to pro-inflammatory cytokines mainly released by the synovial lining, such as TNF-α, IL-6, IL-8, and IL-17, as well as nitric oxide (NO), which increases oxidative stress in the joint. In the synovial fluid, other changes also occur, such as loss of lubricin and increased amounts of fragmented aggrecan because of the catabolism in the articular cartilage.

Microscopically, the main markers of OA cartilage are the loss of collagen type II and proteoglycans, which affects the cartilage ECM structure and disrupts both cartilage homeostasis and biomechanical properties of the tissue [6,7,8,11]. In early OA, spatial organization of the superficial chondrocytes is disturbed. They become dedifferentiated and show increased proliferation and cluster formation [12]. During the disease, the apoptotic death of chondrocytes outweighs cell proliferation, with also an increase in the senescence-related secretory phenotype [13,14]. There is a marked increase in the inflammatory status of both the cartilage and synovial fluid through upregulation of cytokines, such as IL-6, IL-8 and TNFα [15,16]. Recent research has suggested that IL-17 also plays a critical role in OA development, by enhancing the expression of catabolic factors that are involved in the destruction of cartilage [17]. Although interleukin-1 (IL-1) has been a target in OA for many years and some success in trials was achieved recently [18], most murine studies fail to demonstrate protection where the ligands (IL-1α/β) or the receptor (IL-1R) have been knocked out [19]. The major cartilage-degrading enzymes downstream of the inflammatory cascade are aggrecanase-1 and -2 (ADAMTS-4 and ADAMTS-5), which degrade aggrecan, a major ECM proteoglycan in cartilage tissues, and MMP-13, which specifically degrades collagen type II [6,12]. These structural and molecular changes in the OA cartilage are depicted in Figure 1.

According to the most recent guidelines from the Osteoarthritis Research Society International (OARSI) [20], official recommendations for OA treatment include physical measures (education, physical therapy, and weight management) and pharmacological therapies. Despite significant efforts to develop novel biological drugs and cell-based therapies for OA, there is no effective treatment clinically available to date. Small molecules such as corticosteroids and oral NSAIDs (non-steroidal anti-inflammatory drugs), like ibuprofen and diclofenac, are being applied for pain relief and controlling inflammation for OA patients. However, their efficacy in pain relief, let alone cartilage protection, is limited, and accompanied by a high incidence of adverse effects in the long term, such as gastrointestinal and cardiovascular events [3,21]. Surgical interventions, namely total joint replacement/arthroplasty, are indicated only at advanced age given the limited longevity of implants and possibility of revision surgery [22]. Novel drugs clinically tested for OA treatment can be grouped as (1) chondrogenesis inducers, such as bone morphogenetic protein-7 (BMP-7) and recombinant human fibroblast growth factor 18 (FGF-18, sprifermin) [23,24]; (2) anti-inflammatory cytokines, such as IL-1β receptor antagonist and IL-1β receptor antibody [25,26]; and (3) Wnt pathway modulators (lorecivivint) [27,28]. The recent development of nucleic acid-based therapies, such as pDNA, mRNA, siRNA, and aptamers or antisense oligonucleotides (ASO), have shown great potential to modulate pathophysiological pathways at the level of protein synthesis, targeting these pathways across different joint tissues [29,30]. For intra-articular administration, these nucleic acids usually require a carrier to protect them from potential degradation by endonucleases in the extracellular space, as well as to improve retention in the cartilage and avoid rapid clearance from the synovial joint [31].

In comparison with systemic delivery, intra-articular injection is an advantageous alternative for administration of drugs intended to act locally within joint tissues, by achieving high concentrations at the site of administration [32,33]. Apart from the often-serious side effects associated with systemic treatment, drug concentrations in the synovial fluid reach only 23–50% of the levels found in the plasma for most systemically administered drugs [33]. Intra-articular injection of corticosteroid suspensions for OA are commonly used in clinical practice for pain relief, lasting for several weeks after injection [34]. A recent example of approved treatment for OA knee pain based on local injections is ZILRETTA (Flexion Therapeutics), which employs an extended-release microsphere technology combining the corticosteroid triamcinolone acetonide with a poly lactic-co-glycolic acid (PLGA) matrix. In the phase 3 clinical study, ZILRETTA provided significant, clinically meaningful reduction in average daily pain intensity (*p* < 0.0001), compared with saline-solution placebo at week 12 [35].

Intra-articular injections of small molecules alone are often inefficient because of the low retention time and durability of the drug in the joints [36]. Currently, a range of particle-based systems are explored for a targeted and sustained delivery of pharmaceutical drugs, growth factors and small regulatory biomolecules such as siRNAs and ASOs. Overall, these systems are endocytosed without provoking deleterious inflammation. They can enhance half-lives of loaded drugs 10–30 times compared to free drugs [32], thus representing an important tool to improve therapy efficacy.

### 1.2. Nanoscale-Delivery Systems and Polymeric Nanoparticles

Nanocarriers used as drug delivery systems include liposomes, exosomes, micelles, dendrimers, inorganic NPs, and polymeric nanoparticles (PNPs). Encapsulation of small molecules, biological drugs, and nucleic acids in nanocarriers have shown great potential for OA treatment [37]. The main advantages include: (1) superior drug targeting and bioavailability; (2) reduced toxicity and adverse effects; (3) higher drug solubility and stability; (4) prolonged retention time in the cartilage; (5) increased drug efficacy [38,39,40].

Lipid based nanoparticles (LNPs) and liposomes were the first nanocarriers approved by FDA and have many therapeutic applications, such as the current COVID-19 mRNA-LNPs vaccines [41]. They are considered as ideal drug delivery systems due to their excellent biocompatibility and low toxicity, as well as for encapsulating both hydrophilic and hydrophobic drug cargos [42,43]. Despite these promising features, rapid clearance from synovial fluid remains an issue for OA therapy [14]. Other nanocarriers also show important limitations: exosomes display manufacturing challenges [44] and inorganic NPs lack deeper toxicological assessment in literature [45]. Finally, the clinical application of micelles and dendrimers is still hampered by the non-encapsulation of hydrophilic drugs and toxicity concerns [39]. In this review, we focus on another class of delivery vehicles—the polymeric nanoparticles (PNPs).

Polymeric nanoparticles are solid particles that can incorporate hydrophilic and hydrophobic drugs, such as small molecules, proteins, and nucleic acids. They are extensively used in the field of nanomedicine due to their structural versatility, facile synthesis, and higher stability when compared to other nanoparticle types. The major components of PNPs for drug delivery include the presence of a biodegradable polymer–drug linkage (e.g., redox-sensitive disulfide linkages or acid-labile ester bonds)*,* which enables controlled drug release at the target site, and a polymeric carrier with the ability to extend the drug half-life [37,46]. These carriers can be further modified by targeting ligands to achieve cell-specific targeting [47]. The versatility of polymers makes them potentially ideal for fulfilling the requirements of different drug delivery systems and clinical applications [48]. Over 15 types of PNPs have been already approved for clinical use for several disorders and their main benefits include greater protein stability and longer circulation time [49]. In addition, two of the best-selling drugs in the US are polymer-based: Copaxone (glatiramer acetate injection, Teva Pharmaceuticals) for multiple sclerosis, and Neulasta (pegfilgrastim, Amgen) for neutropenia [50].

**Figure 2:**
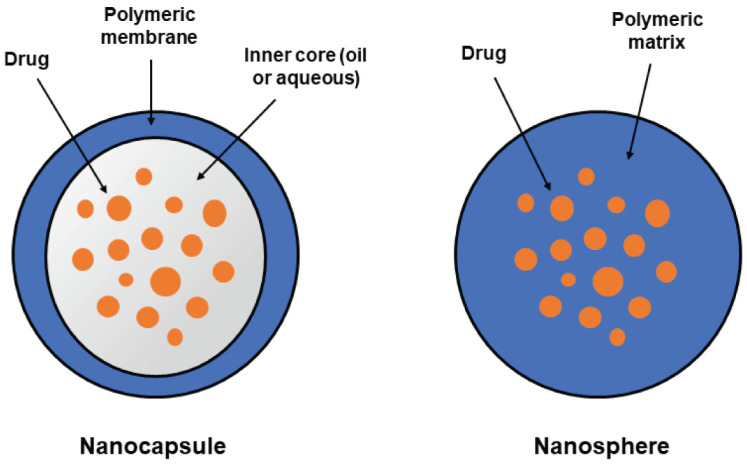
The two main structural forms of polymeric nanoparticles: nanospheres (matrix system) and nanocapsules (reservoir system).

Depending on their origin, PNPs can be classified as natural polymers, such as chitosan, alginate and hyaluronic acid, or synthetic polymers, such as poly(amidoamine) (PAA), poly(lactide-co-glycolide) copolymer (PLGA), and polyethylenimine (PEI) [46]. In addition, they may display two types of structural forms (Figure 2): nanospheres, in which the drug is uniformly dispersed in a polymer matrix, or nanocapsules, in which the drug is loaded within a reservoir core surrounded by a polymeric membrane [51]. In most cases, polymeric NPs are spherical and consist of dense matrices, while self-assembly of amphiphilic conjugates results in the formation of nanocapsules with a hydrophobic core and a hydrophilic shell [48]. For small-scale manufacturing of PNPs, the most common preparation methods are emulsification and solvent evaporation/extraction [52], and the nanoprecipitation technique [53].

Regardless of the nanoparticle architecture or how the payload is incorporated, biodegradability and the controlled release of the cargo remain some of the main features of PNPs in nanomedicine. The most common degradation reactions are by hydrolysis, bio-reduction, enzymatic degradation (e.g., lysozymes, hyaluronidases), and by external factors such as pH and temperature [54]. The degradation rate of polymers is related to factors such as chemical structure, hydrophobicity, and molecular weight [55]. In general, synthetic polymers have longer degradation profiles compared to their natural counterparts, which can be modulated by the incorporation of biodegradable linkers like redox-sensitive disulfide linkages or acid-labile ester bonds.

A summary of the possible strengths and limitations of the use of polymeric nanoparticles as delivery systems, is presented in Table 1 and further discussed in the next sections. In this review, we discuss the current development state of polymeric nanoparticles for drug delivery in OA joint disease by examining the evidence for natural and synthetic polymers as drug carriers to modulate disease pathology and key cellular pathways within the multiple tissues of the joint.

## 2. Methods

Original articles included in this review were obtained from the PubMed database. Search criteria included key words “polymeric OR polymer” AND “nanoparticles OR NPs OR nanospheres OR nanocapsules” AND “delivery” AND “chondrocytes OR synoviocytes OR cartilage OR osteoarthritis”. Relevant studies were found between the year 2006 and 2022. Results were then analyzed according to the following parameters: type of polymer/cargo, chemical functionalization, physical properties (e.g., particle size/zeta potential), study model including route of delivery, and main outcomes. Studies covering other musculoskeletal diseases (e.g., rheumatoid arthritis) or the application of polymeric nanoparticles combined with an additional vehicle (e.g., transfected stem cells or hybrid scaffolds embedded with NPs) were not considered within the scope of this review. In addition, only studies incorporating appropriate negative controls (e.g., free drug and/or empty nanoparticles) for assessment of therapeutical biological effects were included in this review. Until the present (2022), no evidence of completed or ongoing clinical trials was found using the search criteria above.

Studies based on natural (Supplementary Table S1) or synthetic (Supplementary Table S2) polymers are included in two separate sections of this review. The details of the studies were categorized per type of polymer in descending chronological order (most recent publications first by polymer name), followed by a discussion of the major findings. In Table 2, we highlight the main physicochemical properties of the most common PNPs tested as nanocarriers for OA treatment, including surface chemistry, functional groups, and biodegradation mechanism.

## 3. Natural Polymers

Natural polymers include several polysaccharides of plant-based origin that are either positively or negatively charged. They may have linear or branched configurations with amine groups that can be protonated under acidic conditions. Their main advantages as drug delivery vehicles are the biodegradable and biocompatible properties, unique chemical variety and presence of adjustable active sites that confer improved physicochemical properties to different biological applications [47]. As opposed to synthetic polymers, natural polymers can have bioactive effects (e.g., anti-inflammatory) in tissues like cartilage, thus holding an intrinsic therapeutical activity apart from the one provided by the encapsulated drugs [56]. In addition, the inherent antioxidant and anticoagulation effects of polysaccharides ensure a low immunogenicity for in vivo applications [47]. The main limitation in the use of natural polymers is the batch-to-batch variation, because they are derived from natural sources that have a less controlled composition [46]. Natural polymers studied as nano-based delivery systems for OA (Supplementary Table S1) are described below. 

### 3.1. Chitosan

Chitosan is a linear polymer that can be naturally found in the shells of crustaceans and some other organisms. It contains a carbohydrate backbone similar to cellulose that consists of β-1,4-linked D-glucosamine, thus carrying a positive charge from amine groups. In recent years, chitosan has been widely used as a biodegradable and pH-responsive non-viral vector for drug delivery, exhibiting important antioxidant and anti-inflammatory properties [46,57]. Chitosan nanoparticles (CNPs) have been the most common type of natural polymer explored for OA treatment, either alone or combined with other polymers or inorganic compounds, with some examples of preclinical studies so far. 

In a post-traumatic rabbit model of OA, local delivery of CNPs loaded with plasmid DNA encoding IL-1Ra (IL-1 receptor antagonist) was able to significantly reduce severity of histological cartilage lesions compared to plasmids alone, and remarkably sustained the expression of this anti-inflammatory factor in the knee joint synovial fluid for at least 14 days [58]. In primary rabbit chondrocytes, chitosan–pDNA nanoparticles encoding an shRNA targeting MMP-3 and MMP-13 showed greater suppression in mRNA and protein levels, compared to empty NPs and plasmids alone [59]. Glycol chitosan/fucoidan nanogels loaded with the anti-inflammatory peptide KAFAK slowed down cartilage degeneration in a rat OA model, reducing the OARSI score and structural changes in subchondral bone in the KAFAK-loaded NPs to the levels observed in normal rats [60]. The KAFAK-loaded nanoparticles also inhibited expression of inflammatory factors IL-6 and TNF-α in the rat models more than empty NPs and KAFAK alone [60]. 

Chitosan nanoparticles complexed with hyaluronic acid (Ch-HA NPs) were shown to promote efficient targeting by HA binding to the chondrocyte CD44 receptor. For example, Ch-HA NPs loaded with a plasmid encoding for anti-apoptotic CrmA (Cytokine response modifier) significantly decreased cartilage damage and synovial inflammation in rat models when compared to empty NPs, as demonstrated by a downregulation of IL-1β, MMP-3, and MMP-13 expression, and attenuated the loss of collagen type II [61]. Loading of Ch-HA NPs with curcuminoid suppressed inflammation and chondrocyte apoptosis in rat OA knee via repression of the NF-κB pathway [62]. Catabolic activity was also reduced as indicated by lower expression of MMP-1 and MMP-13, whereas collagen type II expression was increased in comparison with direct curcuminoid treatment [62]. Similarly to HA, chondroitin sulfate (CS), a negatively charged glycosaminoglycan present in the natural cartilage tissue, also can be recognized by the CD44 surface marker [63]. Chondroitin sulfate can be functionalized with chitosan nanoparticles by substitution of tripolyphosphate (TPP) for chondrocyte targeting [64]. Indeed, loading of these chitosan-CS NPs with GFP plasmid DNA showed significantly higher transfection efficiency than chitosan-TPP NPs in human arthritic chondrocytes, as well as downregulation of MMP13 expression when chitosan-CS NPs were loaded with MMP13 siRNA [64].

Chitosan nanoparticles have been tested with a variety of functional groups. Grafting of chitosan NPs with hydrophilic SO_3_^−^ groups improved hydration capacity of NPs and provided efficient lubrication under a wide range of loads [65]. Given the wide buffering capacity provided by the amine groups of polyethyleneimine (PEI), chemical functionalization of chitosan NPs with PEI can further improve endosomal escape and protein expression levels of delivered plasmids. Indeed, pEGFP-loaded nanoparticles showed comparable transfection efficiencies with Lipofectamine 2000 in primary rabbit chondrocytes and synoviocytes [66]. In addition, functionalization of a chitosan nanogel with type A endothelin receptor antagonist triggered a decrease in inflammatory and catabolic markers in an OA equine organoid model, after seven days of culture [67]. This therapeutic effect was further enhanced by the combination of a hyaluronic acid nanogel functionalized with a type B1 bradykinin receptor antagonist [67]. 

Furthermore, coating NPs with drugs has shown promise in extending drug effects. The immobilization of anti-IL-6 and anti-TNF-α antibodies on the surface of chitosan nanoparticles significantly ameliorated inflammation in rat models compared to soluble antibodies alone, thus showing a higher reduction in inflammatory cytokine production, fibrosis in the synovial membrane and osteoarticular pain [68]. Intra-articularly injected antioxidant superoxide dismutase (SOD) conjugated to functionalized chitosan (O-HTCC) significantly attenuated mechanical allodynia and suppressed gross histological lesions in OA-induced rats when compared with free SOD treatment [69]. It also enhanced the anti-inflammatory effect and the in vivo antioxidant capacity, as expressed by lower levels of synovial malondialdehyde (MDA) and increased glutathione (GSH) content in the synovial fluid [69]. Another antioxidant, berberine chloride, was loaded in chitosan NPs to evaluate its effect in OA treatment. Results showed significantly higher anti-apoptotic activity than free berberine, and a prolonged retention time in the synovial cavity of at least 96 h [70]. 

### 3.2. Hyaluronic Acid

Hyaluronic acid (HA) is an anionic glycosaminoglycan and a major component of the cartilage extracellular matrix. Due to its lubricating properties, HA plays an important role in maintaining the viscosity and the integrity of the joints. In fact, intra-articular injection of high-molecular-weight HA (HMW, MW > 1000 kDa) is one of the currently clinically available options for OA treatment and was suggested to promote pain relief in knee joints with mild OA [71]. While systemic adverse effects are not predominant, local inflammatory reactions are more common due to degradation of HMW into fragmented low-molecular-weight (LMW, MW < 500 kDa) HA molecules by hyaluronidases [72]. As a drug carrier, hyaluronic acid displays good biocompatibility, biodegradability, high viscoelasticity, and is suitable for cartilage targeting by specific binding to the CD44 receptor [73] highly expressed in articular chondrocytes. 

Self-assembled empty HA nanoparticles (HA-NPs) without any cargo showed a chondroprotective effect by blocking the CD44-NF-κB-catabolic gene axis [74]. While CD44 expression increased in the damaged articular cartilage of patients and mice with OA, intra-articular injection of these self-assembled HA-NPs in OA mice suppressed CD44 expression more effectively than free HMW HA and protected against cartilage destruction [74]. Even in chondrocytes transfected with adenovirus carrying the mouse Cd44 receptor (Ad-Cd44) gene, both CD44 expression and the activation of NF-κB were effectively inhibited by HA-NPs. The downstream expression of catabolic genes was also inhibited, leading to attenuated collagenase activity and lower PGE_2_ production [74]. Hyaluronan nanocapsules loaded with celecoxib also showed superior efficacy over celecoxib alone in attenuating certain osteoarthritis parameters in rat OA models, such as knee swelling, inflammation score and NF-κB pathway activation [75].

### 3.3. Dextran Sulfate

Dextran sulfate is a polymer of sulfated anhydroglucose that is highly water-soluble and has negatively charged branches. Recently, nanoparticles based on dextran sulfate-triamcinolone acetonide (DS-TA) conjugates were tested for their efficacy to treat OA in mice by specifically targeting scavenger receptor class A (SR-A) on activated macrophages [76]. Intra-articular injection of these conjugates not only alleviated the structural damage to cartilage, but also significantly reduced the production of proinflammatory cytokines including IL-1β, IL-6, and TNF-α in the cartilage tissue, compared to untreated OA mice [76]. However, this reduction was not significantly lower than in the groups treated with free TA or empty NPs. 

### 3.4. Elastin

Elastin, like collagen, is a fibrous protein that is present in the extracellular matrix of many connective tissues, such as the cartilage [77]. Elastin-like polypeptides (ELPs) were derived from tropoelastin and comprise multiple copies of the consensus repeat of the native protein, the pentapeptide Val-Pro-Gly-Xaa-Gly (VPGXG) [57]. The repeat units enabled the ELPs to be thermo-responsive, thus having a tunable transition temperature [57]. Thermo-responsive nanoscale vesicles from an elastin-b-collagen like peptide (ELP-CLP) showed burst release behavior by dissociating the vesicles above the unfolding temperature of the CLP domain (>50 °C), indicating the potential of combining hyperthermia treatment for release of encapsulated drugs from appropriately engineered ELP-CLPs [78]. These elastin-like peptides bound collagen and displayed excellent cytocompatibility with chondrocytes and fibroblasts, without starting an inflammatory response as shown by a lack of TNF-α production by activated macrophages [78].

### 3.5. Polyphenols

Polyphenols, such as tannic acid, are organic compounds formed by multiple phenol units which naturally occur in plants as secondary metabolites. Polyphenol nanoparticles have intrinsic antioxidant properties, thus showing great potential as a drug delivery system in the treatment of inflammatory diseases. A boronate-stabilized polyphenol–poloxamer NP (PPNPs) loaded with dexamethasone (DMX) showed ROS scavenging abilities and significantly higher IL-10 secretion in an OA mouse model, compared to free DMX [79]. The DEX-PPNPs also enabled efficient repolarization of M2 macrophages (increased expression of the M2 macrophage marker Arg-1) when compared to saline-injected mice. Angiogenesis, cartilage degradation, and bone erosion were remarkably reduced in DEX-PPNP-treated mice compared to empty nanoparticles and DMX alone [79].

### 3.6. Silk Fibroin

Silk fibroin is a natural fibrous protein derived from the silkworm *Bombyx mori*, as well as by some species of spiders and other arthropods. In addition to their unique mechanical properties and slow biodegradability for retention at the target site, silk fibroin fibers exhibit comparable biocompatibility with other commonly used biomaterials such as polylactic acid and collagen [80]. Silk fibroin nanoparticles loaded with celecoxib or curcumin showed high cell viability of IL-1β-stimulated human articular chondrocytes (hACs) and a stronger decrease in anti-inflammatory activity when compared to free drugs, as demonstrated by significant reduction in IL-6 and RANTES expression [81].

## 4. Synthetic Polymers

Synthetic polymers are produced artificially using polymerization methods. These polymers can be manufactured in a well-defined fashion, therefore presenting a controlled composition and lower batch-to-batch variation in comparison to natural polymers [47]. Common setbacks are related to the lack of biodegradability of water-soluble cationic polymers, such as PEI, PAA, and PDMAEMA, and increased cytotoxicity due to the strong positive charge, resulting from the incorporation of amino functionalities [47]. Despite this, synthetic polymers show great structural versatility, and the amino groups can be further modified to alter these undesirable properties, for example by the incorporation of biodegradable linkers and bioactive functionalities. By incorporation of disulfide linkages or ester bonds into the polymer, the polymeric nanoparticles can be degraded through reduction or hydrolysis of covalent bonds, respectively [46]. Synthetic polymers studied as nano-based delivery systems for OA (Supplementary Table S2) are described below. Table 3 shows recent patents granted to synthetic polymers tested as nanocarriers for OA treatment in the last seven years.

### 4.1. Poly(lactic-co-glycolic Acid) (PLGA)

Polyester PLGA is a biodegradable copolymer of polylactic acid (PLA) and polyglycolic acid (PGA), widely used as a drug delivery system and FDA-approved. Poly(lactic-co-glycolic acid) is the primary polymer used to produce nanoparticle-based delivery systems for intra-articular administration in OA treatment. The main advantages of PLGA as a delivery system include the low toxicity, low immunogenicity, mechanical strength, and chemical flexibility due to the tunable ratio and organization of glycolic and lactic monomers.

Different small molecules tested for OA treatment with anti-inflammatory, antioxidant, and anti-apoptotic effects were explored in combination with PLGA delivery vehicles for improving drug efficacy. A dose up to 10 µM rapamycin loaded in PLGA NPs showed no toxic or inflammatory effects in cultures of primary chondrocytes and synoviocytes isolated from human OA knees [85]. Diacerein (DIA) release from PLGA NPs in cultures of primary rat synoviocytes stimulated with IL-1β and LPS significantly decreased proinflammatory cytokines and matrix-degrading enzymes, compared to empty NPs [86]. In a rat OA model, a single IA injection of DIA-loaded PLGA NPs produced a strong anti-inflammatory response (IL-4 and IL-10) and protected cartilage from degradation, compared to daily oral administration of DIA [86]. Piroxicam (PRX)-loaded PLGA NPs increased drug retention and accumulation in the joint upon IA injection in healthy rats, compared to free PRX injection [87]. Etoricoxib loaded into PLGA-PEG-PLGA triblock copolymer NPs and locally injected into OA rat knees alleviated the OA-related symptoms of subchondral bone, synovium, and cartilage [88]. The expression of matrix degrading enzymes decreased, and aggrecan and collagen type II expression increased, in comparison with etoricoxib alone and empty NPs [88]. The PLGA NPs loaded with the antioxidant Rhein were used to modulate the activity of LPS-stimulated THP-1 macrophages, resulting in the inhibition of IL-1β production and a decrease in ROS production compared to empty NPs [89].

Different areas of the synovial cavity show differences in synovial fluid pH value. Taking advantage of this fact, ammonium bicarbonate (NH_4_HCO_3_) can also be co-loaded into the NPs to enhance drug effectiveness. The porous nature of the PLGA NPs allows the entrance of small molecules such as H_2_O and hydronium ions (H_3_O^+^), which can then react with NH_4_HCO_3_ in the PLGA NP to induce pH neutralization with the production of NH_4_^+^, CO_2_, and H_2_O. This conversion results in the breakdown of the NP shell with sudden release of its cargo. This approach was explored for OA treatment to generate pH-responsive PLGA NPs loaded with Rhein and hyaluronic acid, and the co-loading with NH_4_HCO_3_ resulted in an extracellular burst release behavior and higher cell uptake in comparison with non-responsive PLGA NPs [90,91]. In an OA mouse model, a single IA injection of these pH-responsive NPs was sufficient for a long persistence of hyaluronic acid-loaded PLGA NPs in the knee for 35 days [90]. This injection also promoted a more pronounced reduction in cartilage damage and osteophyte formation than non-responsive nanoparticles.

The coating of PLGA nanoparticles with a targeting ligand is another strategy to improve drug retention and penetration in the cartilage. The PEGylated PLGA NPs loaded with MK-8722, an AMP-activated protein kinase (AMPK) activator, were conjugated with collagen-binding peptide (WYRGRL) [92]. As a result, the PLGA NPs were able to penetrate deep and accumulate preferentially in the cartilage, showing sustained drug release over 48 h in an OA mouse model. Besides, these collagen-targeting PLGA NPs significantly reduced inflammation and promoted higher cartilage repair (95% recovery related to naïve mice), in comparison with non-targeting PLGA NPs [92]. The coating of PLGA NPs with hyaluronic acid (PLGA-HA) was also explored to improve cartilage targeting. The PLGA-HA NPs loaded with near-infrared dye (NIR) showed deeper penetration in ex vivo human explants than unmodified PLGA NPs, and higher accumulation in vivo in the mouse knee following IA injection [93]. The PLGA-HA NPs loaded with Cy3-labelled bovine serum albumin (BSA) showed preferential uptake by chondrocytes in comparison with synoviocytes isolated from human OA patients [94]. When injected in the knee of healthy rats however, these PLGA-HA NPs showed a higher localization to the inner layer of the synovial membrane. The weaker internalization in the cartilage might be explained by the high density of the cartilage extracellular matrix in healthy rats, which becomes less dense after degenerative OA lesions and could facilitate the accessibility of NPs to chondrocytes in damaged cartilage areas. 

Reactive oxygen species (ROS) play an important role in cartilage degradation and chondrocyte death. The PLGA NPs loaded with siRNAs targeting genes involved in ROS production can thus be an efficient strategy to decrease inflammation and oxidative damage in the cartilage. Indeed, PLGA NPs loaded with p47phox siRNA or p66shc siRNA (ROS production inhibitors) were explored in independent studies to assess the therapeutic effects after a single IA injection in OA rats [95,96]. In comparison with control NPs, these siRNA-loaded PLGA NPs promoted a reduction in mechanical allodynia for up to 14–21 days, decrease in proinflammatory cytokines and ROS levels, lower proteoglycan loss [95,96], and even a reduction in the tibial subchondral bony destruction [96].

Nitric oxide (NO) can be used to treat osteoarthritis (OA) by inhibiting inflammation, although a method for controlled release of NO in inflammatory cells is still unavailable. To overcome this challenge, PLGA NPs can be used to encapsulate a photothermal-triggered NO-releasing system, which consists of a Notch1-siRNA conjugated to the surfaces of an NO nanogenerator to specifically inhibit macrophage proliferation [97]. Here the NO generators are hemoglobin (Hb) nanoparticles that act as an NO carrier and can absorb near-infrared light, then converting it into heat to trigger the release of NO. The synergic effect of photothermal therapy, NO and siRNA treatments was demonstrated after IA injection in an OA mouse model under the irradiation of a 650 nm laser. As a result, this system reduced the level of pro-inflammatory cytokines and the macrophage response, and efficiently prevented cartilage erosion and synovial inflammation for 16 days [97].

### 4.2. Polylactic Acid (PLA)

Polylactic acid (PLA) is a biodegradable linear polyester. It is accepted as safe by the FDA and has various medical applications such as in bone implants, screws, and sutures [98]. Polylactic acid-based nanoparticles are promising candidates for drug delivery due to sustained release patterns as they can withstand dissolving over an extensive period. Adenosine-conjugated PLA nanoparticles, in which PEG2000 was bound to adenosine on the 3′, 4′ hydroxyl groups, were synthesized using click chemistry [83]. Extracellular adenosine is an important homeostatic factor for chondrocytes and deficiency of the A2A adenosine receptor (A2AR) directly contributes to OA development [99]. Adenosine nanoparticles prevented OA development in a rat model of PTOA by decreasing knee swelling, markedly reducing fibrillation of the cartilage surface and inhibiting proteoglycan loss, compared to unconjugated NPs [83].

### 4.3. Polycaprolactone (PCL)

Polycaprolactone (PCL) is a hydrophobic, biocompatible, and FDA-approved polyester that is commonly used in polymeric drug delivery systems. The PEG-PCL NPs with cationic diblock copolymer PLL-PCL were conjugated with the EGFR ligand TGFα [82]. The EGFR pathway is critical for maintaining homeostasis of the superficial layer of articular cartilage and its deficiency is related to OA development in knee joints [100]. The retention of these TGFα-NPs in OA joints was remarkably higher than in healthy joints of mice, with no obvious side effects. Besides, the intra-articular administration in an OA mouse model showed that these TGFα-NPs attenuated cartilage degeneration, subchondral bone plate sclerosis, and joint pain by targeting EGFR signaling, compared to free TGFα and unloaded NPs [82].

### 4.4. Poly(hydroxyethyl) methacrylate (pHEMA)

Poly(hydroxyethyl) methacrylate (pHEMA) is a stable and hydrophilic polymer that is widely applied in hydrogels. A common challenge in the field of nanoparticle-based drug delivery is that the ability to engineer particles of variable size is limited. Self-assembling pHEMA nanoparticles with a functionalized hydrophobic side chain of pyridine allowed to vary the particle size range which affected BSA protein retention time in the rat joint space [101]. When using 900 nm pHEMA NPs, the in vivo retention of bovine serum albumin (BSA) delivered to the rat joint was up to 14 days, at significantly higher levels than smaller 500 nm pHEMA NPs and BSA alone [101]. These hydrophobic side chains of pyridine can be further explored to present and deliver IL-1Ra, and this approach was effective in blocking NF-κB activation in a NF-κB inducible reporter cell line [102].

### 4.5. Poly(N-isopropylacrylamide) (pNIPAM)

Poly(N-isopropylacrylamide) (pNIPAM) is a thermo-responsive polymer that has a lower critical solution temperature (LCST) of approximately 32 °C, where the NPs are water soluble below the LCST (hydrophilic phase) and undergo hydrophobic collapse at physiological temperatures. Hollow pNIPAM nanoparticles (i.e., low crosslink density) showed increased encapsulation of the KAFAK peptide (inhibitor of MAPK-activated protein kinase-2) when loading was performed below LCST [103]. These hNPs suppressed IL-6 production for four days in IL-1β stimulated chondrocytes. Moreover, they were successfully delivered in vivo into the joint space of healthy rats, via intra-articular injection, and remained within the joint space for up to seven days [103]. Similarly, these hollow pNIPAM nanoparticles loaded with the MK2-inhibiting YARA peptide also suppressed IL-6 expression in IL-1β stimulated bovine cartilage explants for up to eight days, compared to YARA-loaded solid nanoparticles [104].

### 4.6. Poly(amidoamine) (PAA)

The cationic polymer polyamidoamine (PAA) is usually synthesized by a Michael-type polyaddition and is an excellent scaffold for further chemical functionalization due to the large number of tertiary amino groups on its backbone. Structurally, they can be linear or branched by varying the monomers used for the polymerization. These polymers have a cationic core that allows for efficient condensation of nucleic acids by electrostatic interactions in aqueous solution, and the polymer can have different degrees of branching to further increase delivery efficacy [105,106]. To improve biodegradability, these polymers can be modified with reducible disulfide bonds into the backbone of linear PAA using disulfide-containing cystamine bisacrylamide as reactants in the polyaddition [107]. Indeed, hyperbranched SS-PAA NPs showed dose-dependent transfection of bovine chondrocytes (bCH) and rat tendon derived stem/progenitor cells (rTDSPC) in 2D and 3D cultures [100] at the ratio of mRNA to NPs of 2:50 (*w*/*w*). Transfection in three-dimensional constructs (bCH pellets and tendon-like constructs) suggests the feasibility of this system to native tissues rich in extracellular matrix.

### 4.7. Poly[2-(N,N-dimethylamino)ethyl methacrylate] (PDMAEMA)

The 2-(N,N-dimethylaminoethyl) groups within PDMAEMA polymers possess a tertiary amine that becomes highly protonated and membrane disruptive at endosomal pH values. Its pKa and endosomal escape capacity can be enhanced through copolymerization with the hydrophobic monomer, butyl methacrylate. Colloidally stabilized PEGylated nanoparticles of poly(DMAEMA-co-BMA) can load and very effectively delivery siRNA cytosolically. When surface functionalized with a collagen II binding antibody (mAbCII), these particles could be retained within the joint and sustainably deliver siRNA to block expression of Mmp13 [108]. A single intra-articular injection of this formulation in a mechanical loading model of PTOA mice substantially reduced disease progression, osteophyte formation and expression of MMP-13 in cartilage and synovium, compared to NPs loaded with a nontargeting siRNA sequence [108]. It was also efficient in downregulating expression of genes associated with tissue remodeling, angiogenesis, the innate immune response, and proteolytic enzymes. Furthermore, PDMAEMA-containing nanoparticles were loaded with siRNA against MMP13 and encapsulated within PLGA-based microplates [109]. Following local injection in a PTOA mouse model, microplates retained the nanoparticles in the joint and slowly released them over 28 days, promoting efficient knockdown of MMP13 gene expression and reducing OA phenotype [109].

### 4.8. Poly(aspartic Acid) (PAsp)

Poly(aspartic acid) (PAsp) is a poly(amino acid) with a degradable protein-like amide bond in its backbone and a negatively charged carboxylic acid as a pendant group in each repeating unit. Nanoparticles based on a polyethylene glycol-polyaspartic acid (PEG-PAsp) block co-polymer were used to deliver the cartilage-anabolic runt-related transcription factor (RUNX)1 mRNA in OA knee joints [110]. The OA progression, defined as cartilage degradation, osteophyte formation, and IL-1β expression, was significantly suppressed compared with the control group treated with NPs carrying EGFP mRNA. Furthermore, cell proliferation and expression of the cartilage-anabolic markers SOX9, PCNA and collagen type II were significantly increased in articular chondrocytes of the RUNX1-injected knees [110].

### 4.9. Poly(organophosphazene)

Amphiphilic poly(organophosphazene)s are thermo-responsive nanoparticles with sol-gel transition ability when dissolved in an aqueous solution. As a hybrid system of nanoscaled drug delivery and 3D hydrogel formation, these NPs can be injected as an aqueous solution around room temperature, which then transforms into a hydrogel directly after IA injection due to the body temperature. The corticosteroid triamcinolone acetonide (TCA) was encapsulated by carboxylic acid terminus-functionalized poly(organophosphazene) NPs, by means of the interaction of the hydrophobic core of the polymer with hydrophobic parts of the TCA [111]. Upon intra-articular injection, TCA-encapsulated polymeric NPs turned into a 3D hydrogel after intra-articular injection. A single injection in an OA rat model showed a strong inhibition of the inflammatory profile, illustrated by lower expression of pro-inflammatory cytokines (IL-6, TNF-α) and MMPs (MMP-3, MMP-13) in blood samples, as well as higher anti-inflammatory expression (IL-4, IL-10, and IL-13), compared to the control groups injected with TCA solution [111]. Finally, NP-treated OA knees showed morphological similarity with a normal cartilage, significantly improving protective effects compared with the TCA-solution groups.

### 4.10. Poly(propylene sulfide) (PPS)

Poly(propylene sulfide) (PPS) is an organic polymer synthesized by anionic ring-opening polymerization of the thiirane monomer propylene sulfide. The inherent PPS antioxidant activity has been demonstrated by its ability to scavenge oxygen reactive species (e.g., hydrogen peroxide) and to reduce articular cartilage destruction, following local delivery of drug-free PPS microspheres in a PTOA mouse model [112]. The PPS synthesis by inverse emulsion polymerization lends itself to surface functionalization by covalently grafting peptides to the emulsifier that have affinity for cartilage, such as the collagen II-binding peptide WYRGRL meshwork [113]. Importantly, when this ligand was immobilized on 38 nm PPS nanoparticles (WYRGRL-PPS NPs) and injected intra-articularly into the knees of mice, this system targeted articular cartilage ECM up to 72-fold more than NPs with a scrambled peptide sequence, according to fluorescence quantification of labeled nanoparticles. In comparison, nanoparticles with a mean diameter of 96 nm could not enter the cartilage matrix, which suggests a size limitation given the dense collagen type II network, with a pore size of about 60 nm in the superficial zone that is intertwined with an intricate proteoglycan meshwork [113].

### 4.11. Polyurethane

Polyurethanes are amphiphilic polymers composed of organic units linked by urethane or carbamate bonds (–NH–COO–). They have been widely used in regenerative medicine applications as a drug delivery system due to the biocompatible profile and structural adaptability. For example, amphiphilic polyurethane NPs with pendant amino groups were conjugated with the carboxyl group of kartogenin (PU-KGN NPs) for testing in OA treatment [114]. The intra-articular injection of these NPs in OA rats was shown to be effective in attenuating disease progression and preventing cartilage degeneration, as demonstrated by significant lower OARSI score after 12 weeks of injection and stronger Collagen type II staining than Collagen type I staining, compared to empty NPs and free KGN [114].

### 4.12. Terpolymers

A terpolymer is a polymer that results from copolymerization of three different monomers. Core-shell terpolymer nanoparticles were loaded with anti-inflammatory drugs (celecoxib, tenoxicam, and dexamethasone) as a potential application in osteoarthritis [115]. These amphiphilic nanoparticles composed of vitamin E methacrylate, 1-vinyl-2-pyrrolidone and N-vinylcaprolactam, named as poly(MVE-co-VP-co-VC) NPs, were efficient in reducing the release of inflammatory mediators in LPS-stimulated RAW264.7 macrophages, especially celecoxib and dexamethasone-loaded NPs. On the other hand, tenoxicam-loaded terpolymers caused an overexpression of IL-1β, IL-6, and IL-10 [115]. All these terpolymers proved to be biocompatible in a subcutaneous injection model in rats, with no histological differences compared to PBS control group, two weeks after injection.

## 5. Physicochemical Properties and Tissue Targeting

The capacity for penetration and retention of particles into the cartilage ECM depends largely on their physicochemical properties, including morphology, size, surface charge, and aggregation state [116]. To illustrate the relevance of these properties, the European Nanomedicine Characterization Laboratory (EUNCL) have developed different standard operating procedures for nanomaterial assessment, establishing mean size and polydispersity index as critical quality attributes of a nanoparticle formulation [117]. In this review, we showed that PNPs with a mean volume diameter < 50 nm consistently had efficient penetration into cartilage explants, apart from a high tissue accumulation [82,92,113]. In contrast, larger nanoparticles with a mean diameter of 96 nm may not be able to enter the articular cartilage ECM and are withheld in the superficial zone [113]. Contrary to these concepts, it can be argued that larger nanoparticles display higher resistance to lymphatic clearance in the joints, especially for therapeutical agents that do not need to be delivered intracellularly. For example, larger 900 nm pHEMA NPs showed sustained retention of fluorescent BSA protein in rat stifle joints (≈30% after 14 days), compared to almost no signal from smaller 500 nm NPs [101]. Regarding morphology, the vast majority of polymeric NPs tested for OA treatment are spherical. Compared to irregular shapes, they normally have better cellular uptake due to their isometry, independently of the way they are presented on the cell surface [118].

**Figure 3:**
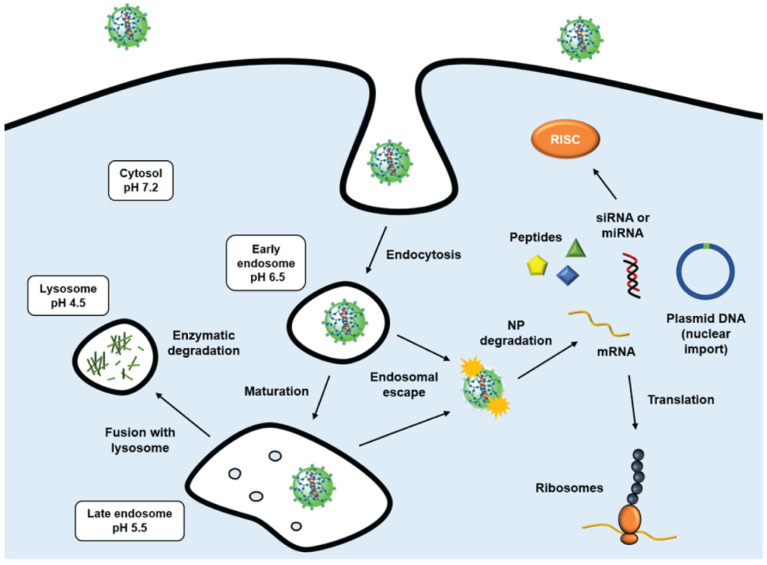
Intracellular trafficking of cationic polymeric nanoparticles. Cell attachment is based on electrostatic interaction between positive charges of nanoparticles and negatively charged groups at the cell surface (e.g., glycosaminoglycans or sialic acid-bearing glycans), followed by the endocytosis of the nanocarriers. Once these NPs are internalized into endosomes, they may escape to the cytosol by the so-called “proton-sponge effect” [123]. According to this model, under the acidic conditions found in endosomes (pH range 6.5 to 5.5), these cationic polymers display buffer abilities by absorbing free protons in the endosome lumen, which in turn leads to an increase in the internal osmotic pressure. High osmotic pressure causes water to shift from the cytosol into the endosome, eventually leading to the burst and release of the entrapped materials. Finally in the cytosol, the nanoparticle is degraded through a biodegradable polymer–drug linkage, which enables controlled release of cargo. If endosomal escape does not occur, the late endosome fuses with the lysosome (pH ≈ 4.5), and the NP is degraded by hydrolytic enzymes. Figure adapted from Figure 1 in reference [124].

Surface charge also directly affects the biological behavior of polymeric nanoparticles. Specifically, cationic nanoparticles display strong interaction with negatively charged nucleic acids or peptides, apart from their ability to bind to cell surfaces and ensure effective uptake through endocytosis. This also influences the choice of the polymeric platform according to the type of cargo and intended release site. In this review, cationic nanoparticles, such as chitosan, PAA, and PDMAEMA, showed great potential for delivery of peptides and nucleic acids intracellularly (Figure 3), as demonstrated by the high uptake through the negatively charged cell membrane [66,84,108]. These polycations can be degraded by incorporated labile linkages such as disulfide bonds that are cleavable within the reducing intracellular environment, or enzymatically cleavable bonds (e.g., peptide sequences) [119]. The magnitude of the surface charge can also impact cartilage penetration, as demonstrated by a higher cartilage uptake and penetration of TGFα-NPs modified with the cationic diblock PLL-PCL (−13.7 mV) than without PLL-PCL (−19.4 mV) [82]. In bovine cartilage explants, these PLL-PCL–modified TGFα-NPs gradually penetrated inside by at least 1 mm by day 6, almost 5-fold more than TGFα-NPs without PLL-PCL. Despite these advantages, cationic PNPs are more prone to interact with serum proteins such as albumin in the synovial fluid, which might affect cell uptake and retention by formation of a protein corona over the NP surface, leading to an increase in particle size [120]. To illustrate this, PRX-loaded NPs consisting of PLGA and cationic Eudragit RL showed higher drug accumulation in joint tissue compared with neutral NPs, with 55% and 83% higher drug remaining 12 and 24 h after dosing, respectively [87]. Possibly the formation of micrometer-sized aggregates, by the interaction between positively charged NPs and anionic HA, might prevent rapid clearance of NPs from the synovial cavity. Surface charge of nanoparticles also have implications in toxicity. For example, when transfecting bovine chondrocytes (bCH) monocultures with cationic SS-PAA NPs, a concomitant toxic effect was observed with increasing mRNA to NP ratios and dosages [84]. This toxic effect can be due to the higher ability of cationic NPs to easily enter cells, in contrast to negatively charged and neutral NPs [121]. The cytotoxic effect might be triggered by mitochondrial function disturbance and cell membrane disruption. It was previously shown that positively charged amino-modified polystyrene nanoparticles caused cell cycle arrest at the G0/G1 phase and decreased cyclin expression, without direct attachment of nanoparticles to chromosome and cytoskeleton [122]. On the other hand, negatively charged carboxylated polystyrene NPs caused much lower cytotoxicity and had no obvious effect on cell cycle [122].

As opposed to passive targeting, which is based on physical interaction between the nanocarriers and the tissue microenvironment, active targeting can lead to a substantial increase in spatial accumulation of nanoparticles in the cartilage. Some studies in this review explored the conjugation of targeting ligands to the surface of PNPs with the aim of promoting its interaction with overexpressed receptors or other cartilage components (Table 2). For example, the hyaluronic acid specificity to receptor CD44, which is overexpressed in chondrocytes from OA cartilage tissues, was explored by HA conjugation onto the surfaces of chitosan and PLGA NPs for enhanced targeting/biodistribution [61,62,94]. Indeed, internalization of HA-covered PLGA NPs was higher in chondrocytes than synoviocytes isolated from human OA patients [94]. Similarly to HA, the chondroitin sulfate (CS) recognition by the CD44 receptor was used to improve uptake of chitosan-CS functionalized nanoparticles, compared to normal chitosan NPs [64]. The collagen-binding peptide (WYRGRL) and mAbCII antibody were also effective ligands that could improve retention time in the joint and ensure sustained release of the cargo. Conjugation of the WYRGRL peptide remarkably improved retention of PPS NPs in the cartilage ECM of mouse knees up to 72-fold more than NPs with a scrambled peptide sequence [113]. Furthermore, PLGA NPs conjugated with the WYRGRL peptide showed higher tissue accumulation compared to empty NPs in mouse femoral heads, with a penetration up to 100 μm [92]. Following 48 h of IA injection in OA mice, 42% of these targeting PLGA NPs remained inside the knees, compared to 18% of the NPs without the targeting peptide. Alternatively, conjugation of mAbCII antibody to poly(DMAEMA-co-BMA) nanoparticles was very efficient to reduce joint clearance, and achieved target-gene silencing in vivo of up to 90% for at least six weeks after IA injection [108].

## 6. Discussion and Future Challenges

The research on polymeric nanoparticles has demonstrated great potential to deliver therapeutical agents that modulate biological factors in OA, such as production of proteolytic enzymes, proinflammatory cytokines, and reactive oxygen species (ROS). Certain polymeric platforms, such as chitosan (natural) and PLGA (synthetic) polymers, stand out for the empirical evidence of regenerative effects and absence of significant toxicity in different types of primary cells, tissue explants, and preclinical OA models. Importantly, altogether these studies showed biological effects in the whole joint, including general reduction in cartilage damage, osseous overgrowth, fibrosis, oxidative stress, pain indicators, and synovial inflammation. Therefore, these nanocarriers can be commonly used soon for clinical trials.

Natural polymers are known to show effective biodegradation and, therefore, they have excellent in vivo biocompatibility. Chitosan and hyaluronic acid undergo enzymatic degradation by lysozymes and hyaluronidases, respectively [54]. On the other hand, synthetic polymers can be engineered to show faster or longer degradation rates. For instance, PLGA NPs are degraded into lactic acid and glycolic acid by hydrolysis of the ester bonds in water, and degradation rate can be adjusted by the lactide/glycolide ratio in the polymer. Given that lactide is more hydrophobic than glycolide, an increase in the proportion of lactide decreases the rate of hydrolytic degradation of the copolymer, with consequent slow release of the encapsulated drug [125]. The PCL nanoparticles have a less acidic character than PLGA and PLA ones and show slower hydrolysis of ester bonds at physiological pH, so that this slower degradation profile prolongs the release of encapsulated drugs [126]. In general, the longer biodegradation profile from PLGA and PCL NPs was useful for sustained release of small molecules and peptides in situ, thus enhancing their concentration/retention in the inflamed joint even after a single IA injection [82,90,93]. Remarkably, TGFα-loaded PEG-PCL NPs showed a substantial increase in the fluorescence signal in OA mouse joints compared to free TGFα at all timepoints over 28 days [82]. In addition, these TGFα NPs were retained in OA joints even longer than those in healthy joints.

Stimuli-responsive nanomedicines can release the therapeutic agent by internal or external triggers, such as temperature, pH, and redox state [127]. Thermo-responsive pNIPAM NPs have a lower critical solution temperature (LCST) of approximately 32 °C, above which (e.g., body temperature) the polymer undergoes hydrophobic collapse and release the cargo. Loaded pNIPAM NPs remained within the joint space of healthy rats for up to seven days, following a single IA injection [103]. Alternatively, encapsulation of PLGA NPs with ammonium bicarbonate (NH_4_HCO_3_) is an effective approach to generate pH-responsive nanoparticles which offer burst release at low pH. Indeed, pH-responsive PLGA NPs loaded with Rhein or hyaluronic acid resulted in an extracellular burst release behavior at a slightly acidic medium pH 5 [89,90]. These pH-responsive PLGA NPs containing HA and NH_4_HCO_3_ induced a fast release of cargo after 24 h and remained in the knee of OA mice for 35 days, following a single IA injection [89]. Disulfide bond-containing PNPs are degraded by glutathione-mediated cleavage of disulfide bonds in the polymer backbone. This degradation relies on the physiological ratio between oxidized (GSSG) and reduced (GSH) forms of glutathione inside and outside the cell [128]. Plasma GSH concentrations are around 1000 times lower than inside the cell (1–10 mM) [129]. Under these highly reducing conditions found in the cytosol, GSH degrades the redox-sensitive disulfide bridges from the polymer and releases the payload. This delivery method can be advantageous for delivery of mRNA or other nucleic acids intracellularly, as previously reported for PAA NPs [84].

Despite the promising results so far, the application of polymeric nanoparticles in OA delivery systems still needs further studies to enhance the cartilage-specific targeting and the long-term retention along with diffusion to the target tissue. While systemic drug delivery is impaired by the absence of blood supply in the cartilage, local delivery is challenged by the rapid lymphatic clearance within synovial joints [37]. Moreover, the cartilage matrix consists of a dense meshwork of collagen type II fibers (50–60%) with a pore size around 60 nm, which poses a clear barrier for full-depth penetration of drugs in the tissue [113]. The remainder of the extracellular matrix is mainly composed of an aggrecan proteoglycan network, containing negatively charged glycosaminoglycan (GAG) side chains which are about 20 nm apart between adjacent aggrecan monomers [130]. Provided that the density of aggrecan increases with depth into the cartilage, their proximity to the collagen meshwork results in a substantial steric hindrance to the penetration of drugs or drug carriers [131].

The longevity and efficiency of NP retention is dependent on trade-offs between cartilage targeting, tissue penetration, and joint clearance of the delivery system. Although small NPs (<100 nm) may have the potential to penetrate deeper into cartilage [131], they are also subject to a faster clearance from the joints, thus compromising biodistribution and sustained drug release in the damaged tissue. The negative charges from GAGs in the cartilage matrix suggests that cationic polymeric nanoparticles would be ideal candidates for OA treatment, by providing favorable electrostatic interactions for targeted drug delivery. However, it can be argued that depletion of proteoglycans at the articular surface in OA disease would reduce the negative charge density of the ECM, thereby reducing electrostatic interactions with cartilage. Indeed, cationic dimethylamine borane (DMAB) nanoparticles experienced a 3-fold reduction in OA tissue retention when compared with healthy cartilage [132].

Additionally, interactions with synovial fluid are a determinant for NP fate in the joint, inducing changes to surface properties and colloidal stability of particles. Synovial fluid can also influence NP interactions with cartilage due to its highly viscous nature, which impedes the transport of macromolecules. For example, after incubation with synovial fluid, polyvinyl alcohol (PVA) NPs did not change their physicochemical properties, whereas polystyrene (PS) NPs experienced significant increase in size and zeta potential [132]. The increased hydrodynamic diameter may reduce the diffusion rate of NPs into the dense ECM, while changes in the zeta potential could impact electrostatic interactions with the ECM. The effect of charge reversal seems to be particularly impacting for cationic NPs, as shown by a significant reduction in DMAB NPs retention in healthy cartilage tissue in the presence of synovial fluid, probably as a result of the diminished electrostatic attraction with the ECM [132].

Nanoparticle properties such as surface charge may also influence off-target accumulation after injection. Nanoparticles that demonstrate stronger biophysical interactions with the cell membrane also tend to show greater cellular uptake. Therefore, cationic NPs usually exhibit greater cell uptake than anionic NPs, due to increased electrostatic interactions with the cell membrane [133]. Another likely fate of NPs, after IA injection in diseased joints, is the phagocytosis by tissue-resident macrophages. Macrophage accumulation in the synovial capsule is a major morphological trait of synovitis, as a result from increased vascular density and inflammatory cell infiltration during OA [134,135]. In addition, macrophages were shown to display increased rates of phagocytosis for positively charged particles [136,137], which is hypothesized to be due to electrostatic interactions with the negatively charged sialic acid groups on the cell membrane [138].

Overall, these findings suggest that the disease state is a determinant in the success of drug delivery to OA cartilage. This may also have an implication for polymeric NPs that use incorporation of reducible disulfide linkages to improve biodegradability, such as poly(amidoamine)s (PAAs) or poly(ethyleneimine)s (PEIs). Bioreducible polymers rely on the glutathione-mediated cleavage of disulfide bonds for drug release inside the target cells, whose efficiency is influenced by the oxidative stress condition in the tissue. For instance, glutathione (GSH) levels in synovial tissue of MIA-induced OA rats were significantly lower than in healthy animals [69]. Furthermore, glutathione content in articular cartilage of adult rats significantly declined with age and continuous loading [139], two important risk factors related to OA development. To ensure controlled release of the payloads under these conditions, NP biodegradability may be tuned by increasing the degree of redox-sensitive disulfide bonds in the polymer backbone.

## 7. Conclusions

Polymeric nanoparticles have shown promising results as drug delivery systems for OA treatment, demonstrating a role as enabling technology to support the OA- and joint inflammation-modulating activity of drug and biomolecular compounds, thus paving the way for future clinical development. Despite recent advances, currently there are no clinical trials investigating polymeric nanoparticles for joint diseases such as OA. The studies carried out so far highlight the importance to evaluate NP interactions with diseased tissue both in vitro and in vivo, as a way to have accurate models that reflect all biochemical and structural changes to the cartilage ECM and synovial fluid. In addition, physicochemical properties such as particle size and surface charge are critical quality attributes that largely influence cartilage targeting, penetration and retention within the joints. The conjugation of specific ligands targeting chondrocytes (e.g., hyaluronic acid) or the collagen type II in the ECM (e.g., collagen II-binding peptide and mAbCII antibody) showed remarkable potential to improve cartilage targeting and reduce joint clearance. Sustained release of small molecules and peptides that act extracellularly in the joint is optimally achieved by using polymers with longer biodegradation profile, such as PLGA and PCL NPs. We have also disclosed the importance to develop multi-functional carriers for OA treatment, including polymers with bioactive properties, responsive to multiple stimuli and with surface modifications. Therefore, possible strategic outlooks include to customize polymeric nanoparticles based on type of cargo, stage of disease, and to identify molecular targets in the joint that are both tissue-specific and stable over the course of disease.

## Figures and Tables

**Table 1:** Summary of possible strengths and limitations in the use of polymeric nanoparticles as drug delivery systems.

Strengths	- High cell uptake and very low immunogenicity. - Excellent transfection efficiency for nucleic acid drugs. - Good water solubility and simple synthesis, ease in scaling-up. - Flexibility, as the same carrier can be used for different payloads. - Incorporation of hydrophilic and hydrophobic drugs. - Possibility to incorporate targeting moieties or dyes for different biological applications. - Prolonged retention due to size limiting vascular and lymphatic clearance.
Limitations	- Batch-to-batch variation (especially natural polymers). - Limited biodegradability for water-soluble synthetic polymers, which often require incorporation of reducible/cleavable disulfide linkages or acid-labile ester bonds. - For cationic polymers, increased cytotoxicity due to the strong positive charge. - Phagocytosis by tissue-resident macrophages.

**Table 2:** Physicochemical properties of the most common polymeric nanoparticles tested for drug delivery in OA treatment. Functional groups listed according to evidence from these studies.

**Natural Polymers**			
**Polymer Name**	**Surface Chemistry** (**Charge/Targeting**)	**Functional Groups/Benefits**	**Biodegradability**
Chitosan	Positive charge (cationic) Active targeting possible: - conjugation of HA onto the NP surfaces - substitution of tripolyphosphate (TPP) by chondroitin sulfate (CS)	- PEG or PVA coating (↑ colloidal stability) - Immobilization of anti-TNF-α and anti-IL-6 Abs at the surface (↑ anti-inflammatory effect) - Grafting with PEI for better transfection efficiency (↑ buffering capacity, ↑ endosomal escape) - Grafting with hydrophilic SO3− groups (↑ hydration capacity) - Grafting with SOD (↑ antioxidant activity)	Enzymatic degradation by lysozymes
Hyaluronic acid (HA)	Negative charge (anionic) Active targeting possible: - natural specificity to CD44 receptor	-	Hydrolysis of β-1,4-glycosidic bonds by hyaluronidases
**Synthetic Polymers**			
Poly(lactic-co-glycolic acid) (PLGA)	Neutral charge Active targeting possible: - surface conjugation of collagen-binding peptide (WYRGRL) - conjugation of HA onto the NP surfaces	- PEG coating (↑ colloidal stability)	Hydrolysis in aqueous media, degradation rate depends on the lactide/glycolide ratio and their molecular weights
Polylactic acid (PLA)	Neutral charge	- PEG coating (↑ colloidal stability) - Surface conjugation of adenosine (ligand for A2A adenosine receptor, anti-inflammatory)	Hydrolysis of the ester bonds, degradation rate depends on molecular weight
Polycaprolactone (PCL)	Neutral charge	- PEG coating (↑ colloidal stability) - Surface conjugation of TGFα (EGFR ligand)	Hydrolysis of the ester bonds, slower degradation at physiological pH
Poly(hydroxyethyl) methacrylate (pHEMA)	Neutral charge	- Incorporation of pyridine hydrophobic side chains to modulate particle size	pH and thermosensitive release: ↑ solubility (drug leakage) at cloud point changes from 28 °C to 39 °C (pH = 6.5)
Poly(N-isopropylacrylamide) (pNIPAM)	Neutral charge	-	Thermosensitive release: phase transition from a water-soluble to insoluble state at temperatures higher than the LCST (>32 °C)
Poly(amidoamine) (PAA)	Positive charge (cationic)	-	Bioreducible NPs, cleavage of disulfide bonds by the intracellular GSH

Abbreviations: Abs: antibodies; EGFR: epidermal growth factor receptor; GSH: reduced glutathione; HA: hyaluronic acid; LCST: lower critical solution temperature; PEG: polyethylene glycol; PEI: polyethyleneimine; PVA: polyvinyl alcohol; SOD: superoxide dismutase.

**Table 3:** Recent patents granted on polymeric nanoparticles and OA (last seven years).

Applicant	Title/Reference	Embodiment for OA Treatment	Experimental Evidence for OA
University of Pennsylvania	Targeting cartilage EGFR pathway for osteoarthritis treatment (WO2022040006A1)	Therapeutic composition comprising a polymeric nanoparticle, a ligand selected to activate an EGFR receptor (e.g., TGFα), and a linker associating the NP and the ligand.	Intra-articular delivery effectively attenuated surgery-induced OA cartilage degeneration, subchondral bone plate sclerosis, and joint pain [82].
New York University	Biodegradable polymeric nanoparticle conjugates and use thereof (WO2017083659A1)	Poly(lactic acid) (PLA) nanoparticle conjugated with adenosine using a polyethylene glycol (PEG) linker.	Intra-articular delivery prevented the development of OA in a rat model of PTOA [83].
20Med Therapeutics B.V.	Nanogels (WO2012165953A1)	Poly(amidoamine) nanoparticles containing disulfide linkages and a biologically active component such as siRNA, miRNA, DNA, (oligo)peptide or proteins.	Transfection of primary chondrocytes and 3D constructs rich in extracellular matrix (bCH pellets and tendon-like constructs) [84].

Abbreviations: bCH: bovine chondrocyte; EGFR: epidermal growth factor receptor; PTOA: post-traumatic osteoarthritis.

## Data Availability

Not applicable.

## Supplementary Materials

The following supporting information can be downloaded at https://www.mdpi.com/article/10.3390/pharmaceutics14122639/s1. Table S1: Efficacy of nanoscale natural polymers in OA; in vitro studies and in vivo models; Table S2: Efficacy of nanoscale synthetic polymers in OA; in vitro studies and in vivo models.
