# Supplementary material for: Polymeric Nanoparticles for Drug Delivery in Osteoarthritis
Source: Pharmaceutics. 2022 Nov 29;14(12):2639. doi: 10.3390/pharmaceutics14122639 (PMC9788411; doi:10.3390/pharmaceutics14122639)
Supplement: Supplementary file 1 [file pharmaceutics-14-02639-s001.zip › Supplementary Table S1.pdf]

Supplementary Table S1. Efficacy of nanoscale natural polymers in OA; *in vitro* studies and *in vivo* models.

**Chitosan**				
**Chemical Functionalization / Physical Properties**	**Cargo**	**Study Model / Route of Delivery**	**Outcomes**	**Ref.**
Substitution of tripolyphosphate (TPP) by chondroitin sulfate (CS). Size: 86 ± 22 nm, spherical; ZP: +6.18 ± 0.02 mV.	GFP pDNA or MMP13 siRNA	*In vitro:* human arthritic chondrocytes.	↑ Transfection and viability of chitosan-CS NPs compared to chitosan-TPP NPs; 80% depression of MMP13 gene expression for transfection with chitosan-CS NPs.	[1]
No modifications. Size: 429 to 733 nm; ZP: +47 to +51 mV.	Endothelin-1 Receptor Antagonist Peptides	*In vitro:* equine cartilage organoid model exposed to interleukin-1 beta (IL-1β).	↓ Serine proteases (HTRA1); ↓ metalloproteinases (MMP1, MMP3 and MMP13); ↓ IL-6; ↓ NO levels; enhanced effect by combination of HA nanogel functionalized with a type B1 bradykinin receptor antagonist.	[2]
Glycol chitosan/fucoidan NPs. Size: 286.3 ± 5.0 nm, irregular shape; ZP: +14.0 ± 0.2 mV.	KAFAK (peptide)	*In vitro:* (IL-1β)-stimulated rat chondrocytes. *In vivo:* ACLT (rats) / IA (single).	*In vitro:* ↓ IL-6 and TNF-α; ↑ chondrogenic markers (collagen II, aggrecan and SOX9). *In vivo:* ↓ IL-6 and TNF-α; ↓ GAG loss; ↓ OARSI score; ↓ osteophyte formation; ↑ tibial subchondral bone density.	[3]
Immobilization of anti-TNF-α and anti-IL-6 Abs at the surface of CNPs. Size: 132.05 ± 2.58 nm, spherical; ZP: +20.07 ± 2.10 mV.	No cargo	*In vitro:* co-culture of hACs with M1 macrophages. *In vivo:* carrageenan (rats) / IA (single).	*In vitro:* ↓ IL-6 and TNF-α; ↑ cell viability; ↑ proliferation. *In vivo:* ↓ IL-6 and TNF-α (stronger than free Abs); ↓ fibrosis; ↓ pain; ↓ inflammation in synovial membrane.	[4]
Grafting of CNPs with hydrophilic SO_3^- groups (↑ hydration capacity). Size: 171.8 nm, spherical; ZP: -25.6 mV.	Aspirin	*In vitro:* primary rat chondrocytes and bovine cartilage.	Cell viability >80% (NP concentration range 0.78 μg/mL to 200 μg/mL); sustained drug release behavior in PBS (pH = 7.4); efficient lubrication under a wide range of loads.	[5]
Conjugation of superoxide dismutase (SOD) to functionalized chitosan (O-HTCC). Size: 236.7 nm, spherical; ZP: +8.3 mV.	No cargo	*In vitro:* rat primary chondrocytes exposed to MIA. *In vivo:* MIA (rats) / IA (1x/week, 4 weeks).	*In vitro:* ↓ intracellular ROS; ↑ cell viability. *In vivo:* ↑ half-life, residence in rat joint cavity and bioavailability; ↓ mechanical allodynia; ↑ antioxidant capacity; ↓ IL-6, TNF-α and IL-1β; ↓ Mankin score.	[6]
Ch-HA NPs. Size: 164.68 ± 14.21 nm, spherical.	Curcuminoid	*In vitro:* rat primary chondrocytes exposed to IL-1β and TNF-α. *In vivo:* ACLT (rats) / IA (1x/week, 4 weeks).	*In vitro:* ↑ proliferation; sustained release; ↓ MMP-1 and MMP-13;↑ collagen II. *In vivo:* ↓ NF-κB (apoptosis); ↓ MMP-1 and MMP-13; ↑ collagen II; ↓ Outerbridge classification; ↓ Mankin score.	[7]

Supplementary Table S1. *Cont.*

**Chemical Functionalization / Physical Properties**	**Cargo**	**Study Model / Route of Delivery**	**Outcomes**	**Ref.**
**Chitosan**				
Ch-HA NPs. Size: 100 to 300 nm, spherical.	CrmA pDNA	*In vitro:* primary rat synoviocytes. *In vivo:* ACLT (rats) / IA (1x/4 weeks, 12 weeks).	*In vitro:* sustained delivery for up to 22 days. *In vivo:* ↓ synovial inflammation (IL-1β, MMP-3 and MMP-13); ↑ collagen II; ↓ OARSI score.	[8]
No modifications. Size: 50 to 400 nm, spherical; ZP: +21.87 ± 1.76 mV.	Berberine chloride (BBR)	*In vivo:* ACLT and DMM (rats) / IA (single).	↑ Anti-apoptotic activity (↓ TUNEL-positive chondrocytes); prolonged retention time in the synovial cavity; ↑ Bcl-2 expression and cartilage protection (↓ caspase-3 and Bax).	[9]
No modifications. Size: 250 to 650 nm, spherical or irregular.	pDNA encoding shRNA targeting MMP-3 and -13	*In vitro:* primary rabbit chondrocytes.	Transfection efficiency of 24.5 ± 1.6%; ↓ MMP-3 and MMP-13 (mRNA and protein levels).	[10]
Grafting of CNPs with LMW PEI. Size: 100 to 300 nm, spherical.	pEGFP	*In vitro:* primary rabbit chondrocytes and synoviocytes.	Transfection efficiency similar to Lipofectamine 2000 and dependent on the weight ratio of NP:DNA; cell viability > 90%.	[11]
No modifications.	Plasmids encoding eGFP, IL-1Ra or IL-10	*In vitro:* primary rabbit chondrocytes and synoviocytes. *In vivo:* PTOA (rabbits) / IA (3x total, every 48h).	*In vitro:* EGFP-loaded NPs can transfect chondrocytes in culture, but not synoviocytes. *In vivo:* transfection efficiency dependent on loaded gene (IL-1Ra increased in the synovial fluid, but no expression for IL-10); ↓ structural damage for IL-1Ra; prolonged retention time in the joint (at least 14 days).	[12]
**Hyaluronic Acid**				
Self-assembled HA NPs prepared by conjugation of free LMW HA backbone with hydrophobic 5β-cholanic acid. Size: 221 ± 1 nm, spherical; ZP: −15.08 ± 0.83 mV.	No cargo	*In vitro:* hACs and mouse primary chondrocytes overexpressing CD44. *In vivo:* DMM (mice) / IA (1x/2 or 4 weeks, 12 weeks).	*In vitro:* ↑ uptake of HA NPs in CD44 overexpression; ↓ IL-1β and PGE2; ↓ catabolic markers (CD44, MMP-3, MMP-13 and COX2); ↓ collagenase activity; ↓ NF-κB. *In vivo:* ↑ retention in knee joint; ↓ CD44 expression; ↓ OARSI score; ↓ subchondral bone plate thickness.	[13]
No modifications. Size: 254.9 to 449.6 nm, spherical; ZP: −36 to −50 mV.	Celecoxib	*In vivo:* MIA (rats) / IA (single).	↓ Knee swelling; ↓ Mankin score; ↓ NF-κB.	[14]
**Dextran Sulfate**				
Self-assembly of DS-TA conjugate in aqueous media. Size: 70 nm, spherical.	Triamcinolone acetonide (TA)	*In vitro:* LPS-activated macrophages (RAW 264.7 cells). *In vivo:* MIA (mice) / IA injections (1x/4 days, 3 weeks).	*In vitro:* ↓ viability of activated macrophages; ↓ IL-1β, IL-6 and TNF-α. *In vivo:* ↓ IL-1β, IL-6 and TNF-α; ↑ GAG; ↓ structural damage (μCT).	[15]

Supplementary Table S1. *Cont.*

**Chemical Functionalization / Physical Properties**	**Cargo**	**Study Model / Route of Delivery**	**Outcomes**	**Ref.**
**Elastin**				
Conjugation of ELP and CLP using CuAAC "Click" Reaction (Thermoresponsive NPs). Size: 100 to 200 nm.	FITC	*In vitro:* LPS-activated RAW 264.7 macrophages, NIH-3T3 cells and ATDC5 cells.	Thermoresponsive burst release at 80 °C (unfolding of the CLP domain); strong retention on collagen substrate; lack of TNF-α production by activated macrophages.	[16]
**Polyphenols**				
Boronate-stabilized polyphenol–poloxamer assembled nanoparticle. Size : 190 nm.	Dexamethasone	*In vitro:* LPS-activated RAW264.7 macrophages. *In vivo:* MIA (mice) / IA (1x/3 days, 30 days).	*In vitro:* ↓ ROS and NO levels; ↑ macrophage M2 repolarization. *In vivo:* ↓ clinical arthritis score; ↑ claw circumference; ↑ kinematics score; ↓ angiogenesis; ↓ structural damage (μCT); ↓ IL-6 and TNF-α; ↑ IL-10.	[17]
**Silk Fibroin**				
No modifications. Size : 110 nm, spherical.	Celecoxib or curcumin	*In vitro:* hACs exposed to IL-1β.	Cell viability >70%; ↓ nitric oxide; ↓ IL-6; ↓ RANTES levels.	[18]

Abbreviations: Abs: antibodies; ACLT: anterior cruciate ligament transection; ATDC5: murine chondrocytes; Ch-HA: chitosan–hyaluronic acid; CLP: short synthetic collagen-like peptides; CNPs: Chitosan nanoparticles; COL2A1: Collagen Type II Alpha 1 Chain; COX-2 cyclooxygenase-2; CrmA Cytokine response modifier; CuAAC: copper(I)-catalyzed alkyne–azide cycloaddition; DMM: destabilization of the medial meniscus; EGFP: enhanced green fluorescent protein; ELP: Elastin-b-collagen-like peptide (ELP); FITC: fluorescein; GAG: glycosaminoglycan; HA: hyaluronic acid; hACs: human articular chondrocytes; HMW HA: high molecular weight hyaluronic acid; IA: intra-articular injection; IL-1Ra: IL-1 receptor antagonist; IM: intramuscular injection; LPS: lipopolysaccharide; MIA: monoidoacetic acid; microCT: microcomputed tomography; MMP13: metalloproteinase-13; NIH-3T3: murine embryotic fibroblasts; NF-κB: factor nuclear kappa B; NO: nitric oxide; O-HTCC: 6-O-2′-hydroxypropyl- trimethylammonium chloride chitosan; OARSI: Osteoarthritis Research Society International; NRS: numerical rating scale; PEI: polyethyleneimine; PGE_2: prostaglandin E2; PTOA: post-traumatic osteoarthritis; RANTES: Regulated on Activation, Normal T cell Expressed and Secreted; ROS: reactive oxygen species; shRNA: small hairpin RNA; SOX9: SRY-Box Transcription Factor 9; TUNEL: Terminal deoxynucleotidyl transferase (TdT) dUTP Nick-End Labeling; ZP: zeta potential.

References

[1] Moghadam, N.A.; Bagheri, F.; Eslaminejad, M.B. Chondroitin sulfate modified chitosan nanoparticles as an efficient and targeted gene delivery vehicle to chondrocytes. *Colloids Surf. B: Biointerfaces* **2022**, *219*, 112786. https://doi.org/10.1016/j.colsurfb.2022.112786

[2] Cullier, A.; Cassé, F.; Manivong, S.; Contentin, R.; Legendre, F.; Garcia-Ac, A.; Sirois, P.; Roullin, G; Banquy, X.; Moldovan, F.; et al. Functionalized Nanogels with Endothelin-1 and Bradykinin Receptor Antagonist Peptides Decrease Inflammatory and Cartilage Degradation Markers of Osteoarthritis in a Horse Organoid Model of Cartilage. *Int J Mol Sci.* **2022**, 23, 8949. https://doi.org/10.3390/ijms23168949

[3] Li, T.; Yang, J.; Weng, C.; Liu, P.; Huang, Y.; Meng, S.; Li, R.; Yang, L.; Chen, C.; Gong, X.. Intra-articular injection of anti-inflammatory peptide-loaded glycol chitosan/fucoidan nanogels to inhibit inflammation and attenuate osteoarthritis progression. *Int. J. Biol. Macromol.* **2021**, *170*, 469–478. https://doi.org/10.1016/J.IJBIOMAC.2020.12.158

[4] Lima, A. C.; Amorim, D.; Laranjeira, I.; Almeida, A.; Reis, R.L.; Ferreira, H.; Pinto-Ribeiro, F.; Neves, N.M. Modulating inflammation through the neutralization of Interleukin-6 and tumor necrosis factor-α by biofunctionalized nanoparticles. *J. Control. Release* **2021**, *331*, 491–502. https://doi.org/10.1016/J.JCONREL.2021.02.001

[5] Yang, L.; Zhao, X.; Zhang, J.; Ma, S.; Jiang, L.; Wei, Q.; Cai, M.; Zhou, F. Synthesis of charged chitosan nanoparticles as functional biolubricant. *Colloids Surf. B: Biointerfaces* **2021**, *206*, 111973. https://doi.org/10.1016/j.colsurfb.2021.111973

[6] Gao, X.; Ma, Y.; Zhang, G.; Tang, F.; Zhang, J.; Cao, J.; Liu, C. Targeted elimination of intracellular reactive oxygen species using nanoparticle-like chitosan-superoxide dismutase conjugate for treatment of monoiodoacetate-induced osteoarthritis. *Int. J. Pharm.* **2020**, *590*, 119947. https://doi.org/10.1016/j.ijpharm.2020.119947

[7] Wang, J.; Wang, X.; Cao, Y.; Huang, T.; Song, D. X.; Tao, H.R. Therapeutic potential of hyaluronic acid/chitosan nanoparticles for the delivery of curcuminoid in knee osteoarthritis and an *in vitro* evaluation in chondrocytes. *Int. J. Mol. Med.* **2018**, *42*, 2604–2614. https://doi.org/10.3892/ijmm.2018.3817

[8] Zhou, P.H.; Qiu, B.; Deng, R.H.; Li, H.J.; Xu, X.F.; Shang, X.F. Chondroprotective effects of hyaluronic acid-chitosan nanoparticles containing plasmid DNA encoding cytokine response modifier A in a rat knee osteoarthritis model. *Cell. Physiol. Biochem.* **2018**, *47*, 1207–1216. https://doi.org/10.1159/000490217

[9] Zhou, Y.; Liu, S.Q.; Peng, H.; Yu, L.; He, B.; Zhao, Q. *In vivo* anti-apoptosis activity of novel berberine-loaded chitosan nanoparticles effectively ameliorates osteoarthritis. *Int. Immunopharmacol.* **2015**, *28*, 34–43. https://doi.org/10.1016/J.INTIMP.2015.05.014

[10] Zhao, J.; Fan, X.; Zhang, Q.; Sun, F.; Li, X.; Xiong, C.; Zhang, C.; Fan, H. Chitosan-plasmid DNA nanoparticles encoding small hairpin RNA targeting MMP-3 and -13 to inhibit the expression of dedifferentiation related genes in expanded chondrocytes. J. Biomed. Mater. Res. A **2014**, *102*, 373–380. https://doi.org/10.1002/jbm.a.34711

[11] Lu, H.; Dai, Y.; Lv, L.; Zhao, H. Chitosan-graft-polyethylenimine/DNA nanoparticles as novel non-viral gene delivery vectors targeting osteoarthritis. *PLoS One* **2014**, *9*, e84703. https://doi.org/10.1371/journal.pone.0084703

[12] Zhang, X.; Yu, C.; XuShi; Zhang, C.; Tang, T.; Dai, K. Direct chitosan-mediated gene delivery to the rabbit knee joints *in vitro* and *in vivo*. *Biochem. Biophys. Res. Commun.* **2006**, *341*, 202–208. https://doi.org/10.1016/J.BBRC.2005.12.171

[13] Kang, L.J.; Yoon, J.; Rho, J.G.; Han, H.S.; Lee, S.; Oh, Y.S.; Kim, H.; Kim, E.; Kim, S.J.; Lim, Y.T.; et al. Self-assembled hyaluronic acid nanoparticles for osteoarthritis treatment. *Biomaterials* **2021**, *275*, 120967. https://doi.org/10.1016/j.biomaterials.2021.120967

[14] El-Gogary, R.I.; Khattab, M.A.; Abd-Allah, H. Intra-articular multifunctional celecoxib loaded hyaluronan nanocapsules for the suppression of inflammation in an osteoarthritic rat model. *Int. J. Pharm.* **2020**, *583*, 119378. https://doi.org/10.1016/J.IJPHARM.2020.119378

[15] She, P.; Bian, S.; Cheng, Y.; Dong, S.; Liu, J.; Liu, W.; Xiao, C. Dextran sulfate-triamcinolone acetonide conjugate nanoparticles for targeted treatment of osteoarthritis. *Int. J. Biol. Macromol.* **2020**, *158*, 1082–1089. https://doi.org/10.1016/j.ijbiomac.2020.05.013

[16] Luo, T.; David, M.A.; Dunshee, L.C.; Scott, R.A.; Urello, M.A.; Price, C.; Kiick, K.L. Thermoresponsive Elastin-b-Collagen-Like Peptide Bioconjugate Nanovesicles for Targeted Drug Delivery to Collagen-Containing Matrices. *Biomacromolecules* **2017**, *18*, 2539–2551. https://doi.org/10.1021/acs.biomac.7b00686

[17] Li, X.; Wang, X.; Liu, Q.; Yan, J.; Pan, D.; Wang, L.; Xu, Y.; Wang, F.; Liu, Y.; Li, X.; et al. ROS-Responsive Boronate-Stabilized Polyphenol–Poloxamer 188 Assembled Dexamethasone Nanodrug for Macrophage Repolarization in Osteoarthritis Treatment. *Adv. Healthc. Mater.* **2021**, *10*, 2100883. https://doi.org/10.1002/adhm.202100883

[18] Crivelli, B.; Bari, E.; Perteghella, S.; Catenacci, L.; Sorrenti, M.; Mocchi, M.; Faragò, S.; Tripodo, G.; Prina-Mello, A.; Torre, M.L. Silk fibroin nanoparticles for celecoxib and curcumin delivery: ROS-scavenging and anti-inflammatory activities in an in vitro model of osteoarthritis. *Eur. J. Pharm. Biopharm*. **2019**, *137*, 37–45. https://doi.org/10.1016/J.EJPB.2019.02.008
