# Supplementary material for: Polymeric Nanoparticles for Drug Delivery in Osteoarthritis
Source: Pharmaceutics. 2022 Nov 29;14(12):2639. doi: 10.3390/pharmaceutics14122639 (PMC9788411; doi:10.3390/pharmaceutics14122639)
Supplement: Supplementary file 1 [file pharmaceutics-14-02639-s001.zip › Supplementary Table S2.pdf]

Supplementary Table S2. Efficacy of nanoscale synthetic polymers in OA; *in vitro* studies and *in vivo* models.

**Poly(lactic-co-glycolic acid) (PLGA)**				
**Chemical Functionalization / Physical Properties**	**Cargo**	**Study Model / Route of Delivery**	**Outcomes**	**Ref.**
Grafting with HA, unmodified PLGA NPs as control. Size: 200 to 228 nm, spherical; ZP: -23 to -15 mV.	FITC or NIR dyes	*In vitro:* C28/I2 human chondrocytes. *Ex vivo:* TNF-stimulated cartilage explants. *In vivo:* healthy mice / IA (single).	*In vitro:* ↑ cell uptake of PLGA-HA NPs after 24h. *Ex vivo:* deeper tissue penetration of PLGA-HA NPs after 48h. *In vivo*: 15 days persistence of NPs in the knee, ↑ accumulation of PLGA-HA NPs.	[1]
Coating with PEG-modified lipid conjugated with collagen-binding peptide (WYRGRL). Size: 25 nm; ZP: -40 mV.	MK-8722 (AMPK activator)	*In vitro*: IL-1β-stimulated mouse femoral heads. *In vivo:* healthy and CIOA mice / IA (5x total, every other day).	*In vitro:* ↓ IL-6 and TNF-α; ↑ tissue accumulation compared to NP without targeting peptide; penetration up to 100μm depth. *In vivo:* sustained drug release over 48h; ↓ TNF-α, IL-1β and NOS2; cartilage recovery to 95% of healthy mice.	[2]
No modifications. Size: 393.8 ± 33.7 nm; ZP: −9.02 ± 2.41 mV.	Rapamycin	*In vitro:* primary chondrocytes and synoviocytes from human knees.	Dose-dependent toxicity for concentrations >10 μM rapamycin NPs (synoviocytes more sensitive than chondrocytes); no inflammation after 24h.	[3]
Non-responsive Rhein-PLGA NPs and pH–responsive Rhein-PLGA NP loaded with ammonium bicarbonate (NH_4HCO_3). Size: 190.7 ± 1.2 nm, spherical; ZP: -22 ± 1.12 mV.	Rhein	*In vitro:* LPS-stimulated THP-1 cells.	↑ Cell uptake and ↓ ROS levels for pH-responsive NPs compared to non-responsive NPs; ↓ IL-6 and TNF-α.	[4]
No modifications. Size: 200 to 320 nm, spherical.	Diacerein (DIA)	*In vitro:* IL-1- and LPS-stimulated rat synoviocytes. *In vivo:* MIA (rats) / IA (single).	*In vitro:* ↓ IL-1, IL-6, TNF-α and COX2; ↓ MMP-3, MMP-13 and ADAMTS-5. *In vivo:* ↓ IL-1, IL-6 and TNF-α; ↑ IL-4 and IL-10; ↓ OARSI score; ↓ cartilage erosions (μCT).	[5]
No modifications. Size: 183.7 ± 72.21 nm; ZP: -41.1 ± 4.81 mV.	p66shc siRNA	*In vivo:* MIA (rats) / IA (single).	↓ Mechanical allodynia for up to 21 days after injection; ↓ proteoglycan loss; ↑ cartilage thickness and number of chondrocytes; ↓ bony surface destruction; ↓ IL-1β, COX2 and TNF-α.	[6]
No modifications. Size: 126 ± 55 nm, spherical; ZP: −23 ± 2 mV.	p47phox siRNA	*In vivo:* MIA (rats) / IA (single).	↓ Mechanical allodynia for up to 14 days after injection; ↓ proteoglycan loss; ↓ calcification of articular cartilage; ↓ ROS levels.	[7]

Supplementary Table S2 | *Cont.*

Poly(lactic-co-glycolic acid) (PLGA)				
**Chemical Functionalization / Physical Properties**	**Cargo**	**Study Model / Route of Delivery**	**Outcomes**	**Ref.**
Photothermal-triggered NO-Hb nanogenerators were encapsulated in PLGA-PEG NPs (as stable NO carriers). Size: 200 nm, spherical; ZP: neutral.	Hb nanoparticles displaying Notch1-siRNA	*In vitro:* LPS-activated RAW 264.7 macrophages. *In vivo:* papain (mice) / IA (1x/3 days, 15 days).	*In vitro:* specific uptake by activated RAW 264.7 cells due to higher Notch1 expression; ↓ TNF-α, IL-1β, IL-6, COX-2 and IL-8. *In vivo:* local accumulation in the inflamed joint and retention for 24 h; ↓ TNF-α, IL-6, and IL-1β; ↓ Notch1 expression; no cartilage erosion (μCT) and synovial inflammation for 16 days; synergistic effect of photothermal therapy, NO and siRNA.	[8]
Non-responsive HA-PLGA NPs and pH–responsive HA-PLGA NP encapsulated with ammonium bicarbonate (NH_4HCO_3). Size: 175 to 202 nm; ZP: −23 to -17 mV.	Hyaluronic acid (HA)	*In vitro:* C28/I2 human chondrocyte cell line. *In vivo:* DMM (mice) / IA (single).	*In vitro:* pH responsive-NPs showed extracellular burst release behavior (pH 5.0); ↑ uptake and viability than non-responsive NPs. *In vivo:* 35 days persistence of NPs in the knee; ↓ osteophyte formation; ↓ cartilage damage.	[9]
PLGA-PEG-PLGA triblock copolymer. Size: 339 nm, spherical; ZP: +1.68 ± 0.85 mV.	Etoricoxib	*In vitro:* IL-1β-stimulated primary chondrocytes from OA patients. *In vivo:* ACLT (rats) / IA (1x/3 weeks, 9 weeks).	*In vitro:* ↓ COX-2, PGE_2 and NO; ↓ MMP-13 and ADAMTS-5. *In vivo:* ↓ OARSI score; ↑ collagen II and aggrecan expression; ↓ MMP-13 and ADAMTS-5, ↑ subchondral bone resorption.	[10]
No modifications. Size: 167 to 207 nm, spherical; ZP: -8.49 mV.	Rhein	*In vitro:* LPS-stimulated THP-1 macrophages.	Cell viability > 80%; ↓ IL-1β; ↓ ROS levels.	[11]
NPs prepared with PLGA and Eudragit RL with the weight ratios of 10:0, 9:1, 8:2, and 7:3. Size: 220 nm, spherical; ZP: +11.5 mV.	Piroxicam (PRX)	*In vivo:* healthy rats / IA (single).	↑ Retention and concentration of the drug in the joint, compared to drug solution and neutrally charged NPs.	[12]
Coating with HA. Size: 335 nm.	BSA conjugated to Cy3 dye	*In vitro:* primary chondrocytes and synoviocytes from OA patients. *In vivo:* healthy rats / IA (single).	*In vitro:* NP uptake begun at 8 h and peaked at 24 h; uptake in chondrocytes > synoviocytes; ↑ TNF-α and IL-1β but lower than inflammatory conditions. *In vivo:* NPs localized to synovial lining, weak internalization in cartilage.	[13]

Supplementary Table S2 | *Cont.*

Chemical Functionalization / Physical Properties	Cargo	Study Model / Route of Delivery	Outcomes	Ref.
**Polylactic acid (PLA)**				
Adenosine-conjugated PLA NPs, in which PEG2000 was bound to adenosine on the 3′,4′ hydroxyl groups. Size: 129 to 144 nm, spherical.	Adenosine	*In vitro:* LPS-activated RAW264.7 macrophages and primary mouse chondrocytes. *In vivo:* PTOA model (rats) / IA (1x/10 days, 8 weeks).	*In vitro:* ↑ cAMP accumulation; ↓ IL-6; ↓ MMP-13; ↓ Collagen X. *In vivo:* ↓ knee swelling; ↓ fibrillation; ↓ proteoglycan loss; ↓ OARSI score and cartilage loss.	[14]
**Polycaprolactone (PCL)**				
Cationic diblock copolymer PLL-PCL introduced into PEG-PCL NPs, and surface conjugation of TGFα (EGFR ligand). Size: 25.93 nm; ZP: −13.7 to −19.4 mV.	No cargo	*Ex vivo:* bovine cartilage explants. *In vivo:* DMM (mice) / IA (1x/ 3 weeks for 2 or 3 months).	*Ex vivo:* ↑ cartilage uptake and penetration of TGFα-NPs with PLL-PCL than without PLL-PCL. *In vivo:* ↑ retention of TGFα-NPs in OA joints than in healthy joints; ↓ Mankin score; ↓ MMP-13 and ADAMTS-5; ↓ subchondral bone sclerosis.	[15]
**Poly(hydroxyethyl) methacrylate (pHEMA)**				
Self-assembled pHEMA polymer with hydrophobic side chains of pyridine. Size: 300 to 700 nm; ZP: +113.3 mV.	IL-1 receptor antagonist (IL-1Ra)	*In vitro:* RAW 264.7 macrophages and NF-κB-luc EU1 cells (NF-κB inducible reporter cell line).	Stable in serum-containing solutions for up to 7 days; ↑ blocking of NF-κB activation for 24 h.	[16]
Self-assembled pHEMA polymer with hydrophobic side chains of pyridine. Size: 500 to 900 nm, spherical.	BSA conjugated with Vivotag®-S 750	*In vivo:* healthy rats / IA (single).	*In vivo:* prolonged retention with larger NPs (up to 14 days), compared to smaller NPs and free BSA.	[17]
**Poly(N-isopropylacrylamide) (pNIPAM)**				
Hollow thermoresponsive pNIPAM NPs. Size: >300 nm; ZP: −25 to −35 mV.	MK2-inhibiting (MK2i) peptide	*In vitro:* IL-1β stimulated primary chondrocytes (fetal bovine knees). *In vivo*: healthy rats / IA (single).	*In vitro:* NPs taken up into chondrocytes within 24 h; cleared from the cells within 6 days; ↓ IL-6 secretion (for up to 4 days). *In vivo:* delivery and retention in the joint space for up to 7 days.	[18]
Hollow thermoresponsive pNIPAM NPs. Size: 293 to 361 nm, spherical; ZP: −5.38 to −8.48 mV.	YARA (peptide)	*In vitro:* RAW 264.7 macrophages. *Ex vivo:* IL-1β stimulated bovine cartilage explants.	*In vitro:* NPs taken up into endosomes of RAW 264.7 macrophages. *Ex vivo:* ↓ IL-6 secretion (for up to 8 days).	[19]
**Poly(amidoamine) (PAA)**				
Hyperbranched and cross-linked PAA NPs. Size: 82 ± 4 nm; ZP: +32 ± 3 mV.	tdTomato mRNA	*In vitro:* primary hBMSCs, hSDSCs, rTDSPCs and bovine chondrocytes.	Transfection efficiency varies according to cell type; dose-dependent transfection and toxicity; optimal mRNA:NP ratio of 2:50 (w/w); toxic effect for w/w ratio 1:50.	[20]

Supplementary Table S2 | *Cont.*

**Chemical Functionalization / Physical Properties**	**Cargo**	**Study Model / Route of Delivery**	**Outcomes**	**Ref.**
**Poly[2-(N,N-dimethylamino)ethyl methacrylate] (PDMAEMA)**				
mAbCII-conjugated PEG-poly(DMAEMA-co-BMA) mixed with non-functionalized PEG-poly(DMAEMA-co-BMA) (1:40 ratio). Size: 100 nm; ZP: neutral.	siRNA against Mmp13	*In vitro:* Chondrogenic ATDC5 mouse cell line. *In vivo:* mechanical loading protocol, 5x/week for 6 weeks (mice) / IA (single).	*In vitro:* >80% knockdown of Mmp13 gene expression; ↑ binding of NPs to exposed Col2 in damaged cartilage. *In vivo:* ↓ MMP-13 expression and protein levels in PTOA joints (cartilage and synovium) after 6 weeks from injection; ↓ IL-1β; OARSI score ↓; protection against meniscal mineralization and osteophyte formation; downregulation of genes related to tissue restructuring, apoptosis, angiogenesis and proteolysis.	[21]
**Poly(aspartic acid) (PAsp)**				
PEG-polyamino acid (Poly{N-[N′-(2-aminoethyl)-2-aminoethyl]aspartamide}) block copolymer (PEG-PAsp). Size: 50.45 to 52.83 nm; ZP: neutral.	Runt-related transcription factor (RUNX)1 mRNA	*In vivo:* medial collateral ligament transection and removal of medial meniscus *(mice)* / IA (1x/3 days, 30 days).	↑ RUNX1 proteins; ↓ cartilage degradation and osteophyte; ↑ cell proliferation; ↑ anabolic factors (SOX9, COL II and PCNA); ↓ IL-1β.	[22]
**Poly(organophosphazene)**				
Carboxylic acid termini-functionalized poly(organophosphazene) (thermosensitive sol-gel transition). Size: 140 nm, spherical; ZP: −4.0 ± 1.6 mV.	Triamcinolone acetonide (TCA)	*In vitro:* mouse fibroblast cell line (NIH3T3). *In vivo:* MIA (rats) / IA (single).	*In vitro:* sustained TCA release for six weeks. *In vivo:* morphological similarity of treated OA knees with healthy cartilage; ↓ MMP-3 and MMP-13; ↓ IL-6 and TNF-α; ↑ IL-4, IL-10, and IL-13.	[23]
**Poly(propylene sulfide) (PPS)**				
Functionalized with collagen II α1 binding peptide (WYRGRL). Size: 38 to 96 nm; ZP: +17.8 ± 3.45 mV.	No cargo	*Ex vivo:* Bovine cartilage explants. *In vivo:* healthy mice / IA (single).	*In vivo:* WYRGRL-PPS NPs targeted cartilage ECM up to 72-fold more than NPs with a scrambled peptide sequence; NPs with a mean size of 96 nm were unable to enter the cartilage matrix.	[24]
**Polyurethane**				
Amphiphilic polyurethane NPs with free amino group for surface conjugation with carboxyl group of KGN (PU-KGN NPs). Size: 25 nm, spherical.	No cargo	*In vitro:* LPS-activated primary rat chondrocytes. *In vivo:* ACTL + DMM (rats) / IA (1x/3 weeks, 9 weeks).	*In vitro:* no proinflammatory effect. *In vivo:* ↓ cartilage degeneration; ↓ OARSI score; ↑ Col II staining; ↓ Col I staining.	[25]

Supplementary Table S2 | *Cont.*

**Terpolymers**				
**Chemical Functionalization / Physical Properties**	**Cargo**	**Study Model / Route of Delivery**	**Outcomes**	**Ref.**
Core-shell terpolymer NPs consisting of vitamin E methacrylate, 1-vinyl-2-pyrrolidone and N-vinylcaprolactam (poly(MVE-co-VP-co-VC)). Size: 110 to 130 nm, spherical; ZP: −1 to −5 mV.	Celecoxib, tenoxicam and dexamethasone	*In vitro:* hACs and LPS-activated RAW264.7 macrophages. *In vivo:* healthy rats / 4x dorsal SC injections.	*In vitro:* celecoxib and dexamethasone-loaded NPs reduced release of inflammatory mediators (NO, TNF-α, IL-1β, IL-6, PGE_2 and IL-10); tenoxicam-loaded NPs reduced release of only NO and PGE_2. *In vivo:* no histological differences after 2 weeks of injection.	[26]

Abbreviations: ACLT: anterior cruciate ligament transection; AMPK: 5' AMP-activated protein kinase; BSA: bovine serum albumin; cAMP: cyclic adenosine monophosphate; CIAO: collagen-induced osteoarthritis; COX-2 cyclooxygenase-2; DMM, destabilization of the medial meniscus; ECM: extracellular matrix; EGFR: epidermal growth factor receptor; FITC: fluorescein; GFP: green fluorescent protein; GAG: glycosaminoglycan; hACs: human articular chondrocytes; Hb: hemoglobin; hBMSCs: human bone marrow-derived mesenchymal stromal cells; hSDSCs: human synovial derived stem cells; IA: intra-articular injection; IM: intramuscular injection; IV: intravenous administration; LPS: lipopolysaccharide; mAbCII: Col2 monoclonal antibody; MIA: Monoidoacetic acid; microCT: microcomputed tomography; MMx: partial medial meniscectomy; NF-κB-luc EU1 cells: B-cell precursor acute lymphoblastic leukemia cell line (EU1) that expresses firefly luciferase in response to NF-κB activation; NIR: near-infrared dye; NO: nitric oxide; NOS2: nitric oxide synthase 2; OARSI: Osteoarthritis Research Society International; PCNA: proliferating nuclear antigen; pDNA: plasmid DNA; PGE_2: prostaglandin E2; PTOA: post-traumatic osteoarthritis; PVA: polyvinyl alcohol; ROS: reactive oxygen species; rTDSPCs: rat tendon derived stem/progenitor cells; SC: subcutaneous injection; shRNA: small hairpin RNA; SOX9: SRY-Box Transcription Factor 9; ZP: zeta potential.

References

[1] Zerrillo, L.; Gigliobianco, M.R.; D'atri, D.; Garcia, J.P.; Baldazzi, F.; Ridwan, Y.; Fuentes, G.; Chan, A.; Creemers, L.B.; Censi, R.; et al. PLGA Nanoparticles Grafted with Hyaluronic Acid to Improve Site-Specificity and Drug Dose Delivery in Osteoarthritis Nanotherapy. *Nanomaterials* **2022**, *12*, 2248. https://doi.org/10.3390/nano12132248

[2] Ai, X.; Duan, Y.; Zhang, Q.; Sun, D.; Fang, R.H.; Liu-Bryan, R.; Gao, W.; Zhang, L. Cartilage-targeting ultrasmall lipid-polymer hybrid nanoparticles for the prevention of cartilage degradation. *Bioeng. Transl. Med.* **2021**, *6*, e10187. https://doi.org/10.1002/btm2.10187

[3] Pape, E.; Parent, M.; Pinzano, A.; Sapin-Minet, A.; Henrionnet, C.; Gillet, P.; Scala-Bertola, J.; Gambier, N. Rapamycin-loaded Poly(lactic-co-glycolic) acid nanoparticles: Preparation, characterization, and *in vitro* toxicity study for potential intra-articular injection. *Int. J. Pharm.* **2021**, *609*, 121198. https://doi.org/10.1016/J.IJPHARM.2021.121198

[4] Hu, B.; Gao, F.; Li, C.; Zhang, B.; An, M.; Lu, M.; Liu, Y.; Liu, Y. Rhein laden pH-responsive polymeric nanoparticles for treatment of osteoarthritis. *AMB Express* **2020**, *10*, 158. https://doi.org/10.1186/s13568-020-01095-3

[5] Jung, J.H.; Kim, S.E.; Kim, H.J.; Park, K.; Song, G.G.; Choi, S.J. A comparative pilot study of oral diacerein and locally treated diacerein-loaded nanoparticles in a model of osteoarthritis. *Int. J. Pharm.* **2020**, *581*, 119249. https://doi.org/10.1016/j.ijpharm.2020.119249

[6] Shin, H.J.; Park, H.; Shin, N.; Shin, J.; Gwon, D.H.; Kwon, H.H.; Yin, Y.; Hwang, J.A.; Hong, J.; Heo, J.Y.; et al. P66shc siRNA nanoparticles ameliorate chondrocytic mitochondrial dysfunction in osteoarthritis. *Int. J. Nanomedicine* **2020**, *15*, 2379–2390. https://doi.org/10.2147/IJN.S234198

[7] Shin, H.J.; Park, H.; Shin, N.; Kwon, H.H.; Yin, Y.; Hwang, J.A.; Kim, S.I.; Kim, S.R.; Kim, S.; Joo, Y.; et al. P47phox siRNA-loaded PLGA nanoparticles suppress ROS/oxidative stress-induced chondrocyte damage in osteoarthritis. *Polymers* **2020**, *12*, 443. https://doi.org/10.3390/polym12020443

[8] Chen, X.; Liu, Y.; Wen, Y.; Yu, Q.; Liu, J.; Zhao, Y.; Liu, J.; Ye, G. A photothermal-triggered nitric oxide nanogenerator combined with siRNA for precise therapy of osteoarthritis by suppressing macrophage inflammation. *Nanoscale* **2019**, *11*, 6693–6709. https://doi.org/10.1039/c8nr10013f

[9] Zerrillo, L.; Que, I.; Vepris, O.; Morgado, L.N.; Chan, A.; Bierau, K.; Li, Y.; Galli, F.; Bos, E.; Censi, R.; et al. pH-responsive poly(lactide-co-glycolide) nanoparticles containing near-infrared dye for visualization and hyaluronic acid for treatment of osteoarthritis. *J. Control. Release* **2019**, *309*, 265–276. https://doi.org/10.1016/J.JCONREL.2019.07.031

[10] Liu, P.; Gu, L.; Ren, L.; Chen, J.; Li, T.; Wang, X.; Jang, J.; Chen, C.; Sun, L. Intra-articular injection of etoricoxib-loaded PLGA-PEG-PLGA triblock copolymeric nanoparticles attenuates osteoarthritis progression. *Am. J. Transl. Res.* **2019**, *11*, 6775–6789. http://www.ncbi.nlm.nih.gov/pubmed/31814887

[11] Gómez-Gaete, C.; Ferreira, F.; Bustos, P.; Mennickent, S.; Castillo, D.; Chávez, C.; Novoa, P.; Godoy, R. Optimization of rhein-loaded polymeric nanoparticles using a factorial design and evaluation of the cytotoxic and anti-inflammatory effects. *Drug Dev. Ind. Pharm.* **2018**, *44*, 1285–1294. https://doi.org/10.1080/03639045.2018.1445263

[12] Kim, S.R.; Ho, M.J.; Kim, S.H.; Cho, H.R.; Kim, H.S.; Choi, Y.S.; Choi, Y.W.; Kang, M.J. Increased localized delivery of piroxicam by cationic nanoparticles after intra-articular injection. *Drug Des. Devel. Ther.* **2016**, *10*, 3779–3787. https://doi.org/10.2147/DDDT.S118145

[13] Riffault, M.; Six, J.L.; Netter, P.; Gillet, P.; Grossin, L. PLGA-Based Nanoparticles: A Safe and Suitable Delivery Platform for Osteoarticular Pathologies. *Pharm. Res.* **2015**, *32*, 3886–3898. https://doi.org/10.1007/s11095-015-1748-5

[14] Liu, X.; Corciulo, C.; Arabagian, S.; Ulman, A.; Cronstein, B.N. Adenosine-Functionalized Biodegradable PLA-b-PEG Nanoparticles Ameliorate Osteoarthritis in Rats. *Sci. Rep.* **2019**, *9*, 7430. https://doi.org/10.1038/s41598-019-43834-y

[15] Wei, Y.; Luo, L.; Gui, T.; Yu, F.; Yan, L.; Yao, L.; Zhong, L.; Yu, W.; Han, B.; Patel, J.M.; et al. Targeting cartilage EGFR pathway for osteoarthritis treatment. *Sci. Transl. Med.* **2021**, *13*, eabb3946. https://doi.org/10.1126/SCITRANSLMED.ABB3946

[16] Agarwal, R.; Volkmer, T.M.; Wang, P.; Lee, L.A.; Wang, Q.; García, A.J. Synthesis of self-assembled IL-1Ra-presenting nanoparticles for the treatment of osteoarthritis. *J. Biomed. Mater. Res. A* **2016**, *104*, 595–599. https://doi.org/10.1002/jbm.a.35601

[17] Singh, A.; Agarwal, R.; Diaz-Ruiz, C.A.; Willett, N.J.; Wang, P.; Lee, L.A.; Wang, Q.; Guldberg, R.E.; García, A.J. Nanoengineered particles for enhanced intra-articular retention and delivery of proteins. *Adv. Healthc. Mater.* **2014**, *3*, 1562–1567. https://doi.org/10.1002/adhm.201400051

[18] Deloney, M.; Smart, K.; Christiansen, B.A.; Panitch, A. Thermoresponsive, hollow, degradable core-shell nanoparticles for intra-articular delivery of anti-inflammatory peptide. *J. Control. Release* **2020**, *323*, 47–58. https://doi.org/10.1016/J.JCONREL.2020.04.007

[19] McMasters, J.; Poh, S.; Lin, J. B.; Panitch, A. Delivery of anti-inflammatory peptides from hollow PEGylated poly(NIPAM) nanoparticles reduces inflammation in an *ex vivo* osteoarthritis model. *J. Control. Release* **2017**, *258*, 161–170. https://doi.org/10.1016/j.jconrel.2017.05.008

[20] Sturm, L.; Schwemberger, B.; Menzel, U.; Häckel, S.; Albers, C.E.; Plank, C.; Rip, J.; Alini, M.; Traweger, A.; Grad, S; et al. *In vitro* evaluation of a nanoparticle-based mRNA delivery system for cells in the joint. *Biomedicines* **2021**, *9*, 794. https://doi.org/10.3390/biomedicines9070794

[21] Bedingfield, S.K.; Colazo, J.M.; Yu, F.; Liu, D.D.; Jackson, M.A.; Himmel, L.E.; Cho, H.; Crofford, L.J.; Hasty, K.A.; Duvall, C.L. Amelioration of post-traumatic osteoarthritis via nanoparticle depots delivering small interfering RNA to damaged cartilage. *Nat. Biomed. Eng.* **2021**, *5*, 1069–1083. https://doi.org/10.1038/s41551-021-00780-3

[22] Aini, H.; Itaka, K.; Fujisawa, A.; Uchida, H.; Uchida, S.; Fukushima, S.; Kataoka, K.; Saito, T.; Chung, U.; Ohba, S. Messenger RNA delivery of a cartilage-anabolic transcription factor as a disease-modifying strategy for osteoarthritis treatment. *Sci. Rep.* **2016**, *6*, 1–12. https://doi.org/10.1038/srep18743

[23] Seo, B.B.; Kwon, Y.; Kim, J.; Hong, K.H.; Kim, S.E.; Song, H.R.; Kim, Y.M.; Song, S.C. Injectable polymeric nanoparticle hydrogel system for long-term anti-inflammatory effect to treat osteoarthritis. *Bioact. Mater.* **2021**, *7*, 14–25. https://doi.org/10.1016/J.BIOACTMAT.2021.05.028

[24] Rothenfluh, D.A.; Bermudez, H.; O'Neil, C.P.; Hubbell, J.A. Biofunctional polymer nanoparticles for intra-articular targeting and retention in cartilage. *Nat. Mater.* **2008**, *7*, 248–254. https://doi.org/10.1038/nmat2116

[25] Fan, W.; Li, J.; Yuan, L.; Chen, J.; Wang, Z.; Wang, Y.; Guo, C.; Mo, X.; Yan, Z. Intra-articular injection of kartogenin-conjugated polyurethane nanoparticles attenuates the progression of osteoarthritis. Drug Deliv. **2018**, *25*, 1004–1012. https://doi.org/10.1080/10717544.2018.1461279

[26] Pontes-Quero, G.M.; Benito-Garzón, L.; Pérez Cano, J.; Aguilar, M.R.; Vázquez-Lasa, B. Modulation of Inflammatory Mediators by Polymeric Nanoparticles Loaded with Anti-Inflammatory Drugs. *Pharmaceutics* **2021**, *13*, 1–21. https://doi.org/10.3390/pharmaceutics13020290
